# Anions as Dynamic Probes for Ionic Liquid Mixtures

**DOI:** 10.1021/acs.jpcb.0c00026

**Published:** 2020-03-18

**Authors:** Maria Enrica Di Pietro, Franca Castiglione, Andrea Mele

**Affiliations:** † Department of Chemistry, Materials and Chemical Engineering “G. Natta”, Politecnico di Milano, Piazza L. da Vinci 32, 20133 Milano, Italy; ‡ Istituto di Scienze e Tecnologie Chimiche (SCITEC-CNR), Via A. Corti 12, 20133 Milano, Italy

## Abstract

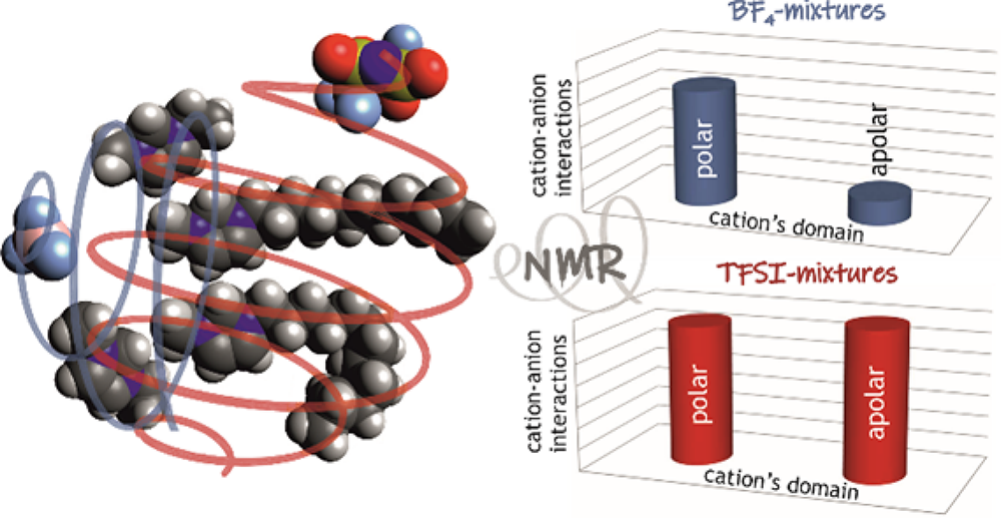

Ionic liquid (IL) mixtures have been proposed as a viable alternative to rationally fine-tune the physicochemical properties of ILs for a variety of applications. The understanding of the effects of mixing ILs on the properties of the mixtures is however only in the very early stages. Two series of ionic liquid mixtures, based on the 1-ethyl-3-methylimidazolium and 1-dodecyl-3-methylimidazolium cations, and having a common anion (tetrafluoroborate or bis(trifluoromethylsulfonyl)imide), have been prepared and deeply characterized via multiple NMR techniques. Diffusion and relaxation methods combined with 2D ion–ion correlation (nuclear Overhauser enhancement) experiments have been used for a better understanding of the interplay between dynamics and structure of IL mixtures. A crucial role of the anion in driving the mixture’s behavior emerged, making them important “dynamic probes” for gaining information of the polar and nonpolar regions of ionic liquids and their mixtures.

## Introduction

Ionic liquids (ILs) are liquid materials that consist entirely of ions and usually melt below 100 °C.^1,2^ ILs have potentially a number of advantages, including very low vapor pressure, low flammability, high electrochemical stability, large liquid ranges, and generally good thermal stability.^3^ This has drawn considerable interest both among the academic community and in terms of industrial applications.^2,4−6^

A peculiar feature of ILs is the opportunity to fine-tune their properties for a given application by mixing and matching the cationic and anionic constituents and by altering and functionalizing the structure of the anion, cation, or both.^1^ This has led to large libraries of ILs synthesized with the aim of achieving specific properties.^3^ Next to the amount of synthetic work needed, this approach is hindered by the lack of toxicological characterization of the new ILs, which slows down, if not prevent, their industrial application. An alternative approach to increase such synthetic variety is to mix two, or more, toxicologically well-characterized ILs together to produce a range of new systems possessing combination of properties.^1,7−9^

Mixtures of ILs containing different cations and anions only started receiving significant attention in the past 10 to 15 years.^1,3,7,9−33^ For a rational fine-tuning of the properties of IL mixtures, the understanding of structural organization, motion, and intermolecular interactions at the molecular level is pivotal to tailor the system for a given application.^11,30^

Here, we focus on the structure and dynamics of mixtures based on 1-alkyl-3-methylimidazolium salts ([C*_n_*mim][X]) with a common anion. We adopt the nomenclature based upon the number of components (instead of the number of constituents); hence, we refer to the mixtures as binary mixtures.^1,8^ Keeping the anion constant, two cations with a short chain [C_2_mim]^+^ and a long chain [C_12_mim]^+^ were chosen as representatives of quite different dynamic and structural behaviors. The choice is intended to maximize the “anomalies” of the mixtures by combining an IL with no nanostructure, [C_2_mim][X], with one containing the amphiphilic cation, [C_12_mim]^+^. Indeed, the available literature seems to converge upon the conclusion that most IL mixtures display close to ideal mixing behavior and deviations may for instance occur in case of substantial differences in the ion sizes, as in the present study.^10,12,24,30^ To gather detailed information on the modulation of the properties of the mixture, various molar ratios of the two components were studied, ranging from compositions with a marked excess of one component to equimolar mixtures. As for the anion, we selected two fluorinated species, bistriflimide, [TFSI]^−^ (systematically known as bis(trifluoromethane)sulfonimide and often referred to as [NTf_2_]^−^), and tetrafluoroborate, [BF_4_]^−^.

To the best of our knowledge, no investigation has been carried out on such mixtures in terms of dynamics. On the contrary, accurate recent studies reported on the (nano)structure of [C_2_mim][C_12_mim][TFSI] mixtures investigated by small-angle X-ray and neutron scattering measurements, as well as molecular dynamics (MD) simulations.^3,30^ At low concentrations of [C_12_mim][TFSI], isolated pseudospherical aggregates of [C_12_mim]^+^ were found within the polar network composed of the charged imidazolium heads and the [TFSI]^−^ ions. The number of these aggregates grows with increasing [C_12_mim][TFSI] concentration and coalesces to form a continuous, nonpolar subphase. At high concentrations of [C_12_mim][TFSI], the liquid structure is similar to that of pure [C_12_mim][TFSI] and the system can be described as a bicontinuous network of continuous polar and nonpolar domains. Very recently, also binary mixtures [C_2_mim]*_x_*[C_8_mim]_1–*x*_[BF_4_] were investigated by X-ray scattering and MD simulations.^20^ Similar to what found for [C_2_mim][C_12_mim][TFSI] mixtures, a disruption of the bicontinuous morphology and a transition to more isolated aggregates upon dilution with [C_2_mim][BF_4_] were observed. Analogous structural behaviors characterize also [C_2_mim][C_6_mim][TFSI] and [C_2_mim][C_10_mim][TFSI] mixtures, where the ethyl groups of [C_2_mim]^+^ turn out not to enter into the nonpolar domains formed by the hexyl or decyl groups.^10,28^ Contrarily, when two cations are combined with chains of more similar size, as in [C_6_mim][C_10_mim]Cl and [C_4_mim][C_10_mim][TFSI] mixtures, there is no differentiation between domains built up only by the short (butyl or hexyl) and the long (decyl) chains.^10,34^ The different behavior of such mixtures has been explained by assuming that the ethyl chains cannot enter into van der Waals dispersion interactions sufficiently to overcome those between the hexyl, decyl, or dodecyl chains, and hence, the separation is maintained. On the other hand, the hexyl and decyl chains of [C_6_mim][C_10_mim]Cl and the butyl and decyl chains of [C_4_mim][C_10_mim][TFSI] can both enter into van der Waals dispersion interactions allowing their intimate mixing.

The focus of the previous studies is mainly the cation–cation mutual arrangement. From our viewpoint, what deserves now a closer look is the role played by the anion in the dynamic behavior and structural organization of such mixtures. To shed light on this aspect, we selected two anions that are known to interact with a different strength to the imidazolium head of [C*_n_*mim]^+^ cations: a soft anion, [TFSI]^−^, and a relatively hard one, [BF_4_]^−^.^35^ Indeed, from electrospray ionization mass spectrometry data, it was possible to measure the relative strength of anion–cation interactions inside different ILs differentiating between two classes: anions tightly coordinated to the cationic moiety, that include [BF_4_]^−^, and anions loosely interacting with the alkylimidazolium species, such as [TFSI]^−^.^35^ Although pure TFSI-based ILs are known to be in general less structured materials than the analogous BF_4_-based ILs, the effect of the anion on dynamics and structure of [C_12_mim][C_2_mim][X] mixtures is still an unexplored issue.

The aim of the present work is then to contribute to filling the knowledge gap on IL mixtures in terms of dynamics and to widening the understanding of their nanostructure at the molecular level, with a special focus on the role of the anion.

To these end, multiple nuclear magnetic resonance (NMR) techniques are applied. NMR stands out as a competitive and well-established method to get insights into structural and dynamic properties of ILs at the molecular level.^36,37^ We present in the following a comprehensive NMR study of [C_12_mim][C_2_mim][BF_4_] and [C_12_mim][C_2_mim][TFSI] mixtures. Spin–lattice and spin–spin NMR relaxation and pulsed field gradient spin-echo NMR are applied to gather information on the rotational and translational motion of the individual cations and anions in the mixture, while homo and heteronuclear nuclear Overhauser enhancement (NOE) correlations make it possible to detect the intermolecular interactions that are descriptors of the local structure of the mixtures.

## Experimental Methods

### Samples

**Figure 1:**
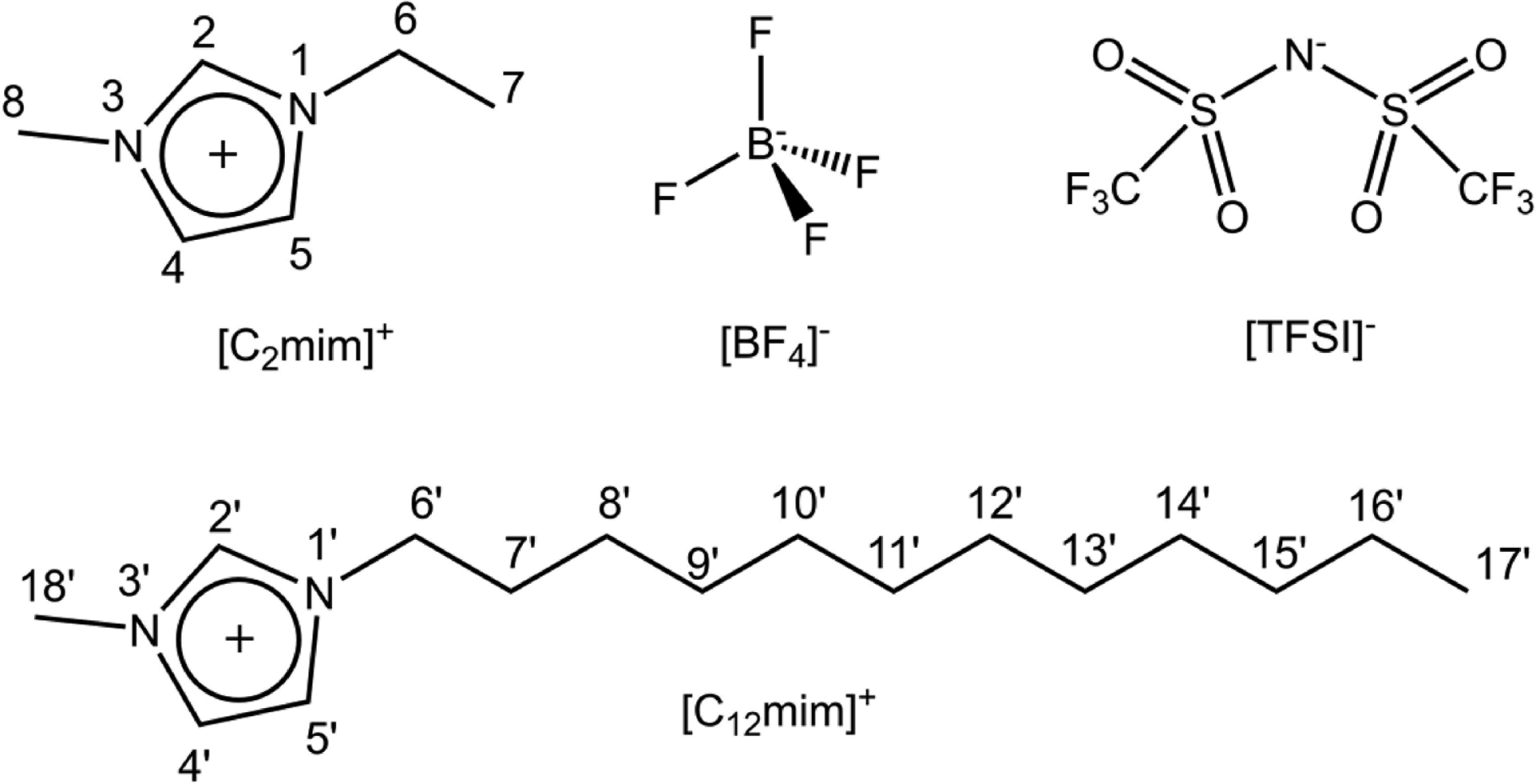
Structure and numbering of the cations and anions constituting the ionic liquids used in this work.

The ionic liquids 1-ethyl-3-methylimidazolium tetrafluoroborate (99%), 1-ethyl-3-methylimidazolium bis(trifluoromethylsulfonyl)imide (99.5%), 1-dodecyl-3-methylimidazolium tetrafluoroborate (>98%), and 1-dodecyl-3-methylimidazolium bis(trifluoromethylsulfonyl)imide (98%) were purchased from Iolitec. The chemical structures of all cations and anions are depicted in Figure 1. Mixtures were prepared by mixing the pure components in the appropriate proportions. The six binary mixture series investigated in this work are listed in Table 1, together with the four pure components. They all display the general formula [C_12_mim]_1–*x*_[C_2_mim]*_x_*[TFSI] and [C_12_mim]_1–*x*_[C_2_mim]*_x_*[BF_4_], with *x* = 0.1, 0.5, 0.9. Corresponding short names are also indicated, which will be used in the following for the sake of simplicity. After proper stirring, samples were dried under vacuum and transferred in 5 mm NMR tubes. The tubes, equipped with a capillary containing DMSO-*d*_6_, were immediately flame-sealed after transferring the samples. While the water concentrations were not explicitly measured in the samples, the absence of a detectable water signal in the NMR spectra indicated that the water content is negligible.^38^

**Table 1:** Samples Used in this Work

short name	description
BF_4_-10:0	[C_12_mim][BF_4_]
BF_4_-9:1	[C_12_mim]_0.9_[C_2_mim]_0.1_[BF_4_]
BF_4_-5:5	[C_12_mim]_0.5_[C_2_mim]_0.5_[BF_4_]
BF_4_-1:9	[C_12_mim]_0.1_[C_2_mim]_0.9_[BF_4_]
BF_4_-0:10	[C_2_mim][BF_4_]
TFSI-10:0	[C_12_mim][TFSI]
TFSI-9:1	[C_12_mim]_0.9_[C_2_mim]_0.1_[TFSI]
TFSI-5:5	[C_12_mim]_0.5_[C_2_mim]_0.5_[TFSI]
TFSI-1:9	[C_12_mim]_0.1_[C_2_mim]_0.9_[TFSI]
TFSI-0:10	[C_2_mim][TFSI]

### NMR Methods

^1^H and ^19^F NMR experiments were carried out on a Bruker Avance 500 spectrometer equipped with a 5 mm pulsed-field z-gradient QNP four nuclei switchable probe. HOESY experiments were carried out on a Bruker NEO 500 spectrometer equipped with a 5 mm pulsed-field z-gradient BBFO probe. For each sample, the probe was carefully tuned, and the 90° pulses were evaluated. The sample temperature was set and controlled using a variable temperature control unit using air gas flow. Variable temperature experiments were performed changing the temperature from 300 to 325 K.

T_1_ and T_2_ relaxation measurements were performed with the inversion recovery (IR) and the Carr–Purcell-Meiboom–Gill (CPMG) pulse sequences, respectively. All relaxation measurements were carried out with relaxation delays at least five times T_1_. Cation and anion relaxation times were measured independently by carrying out IR and CPMG experiments in the ^1^H and ^19^F frequency domains, respectively.

The spin–lattice relaxation rates were measured using data matrices of 8192 (t_2_) × 16 (t_1_) and 32768 (t_2_) × 16 (t_1_) complex data points for ^1^H and ^19^F, respectively. Proton T_1_ experiments were carried out over a spectral width of 14 ppm for various delay time τ, ranging from 0.01–5 to 0.05–20 s (for BF_4_-samples) and from 0.05–7 to 0.05–40 s (for TFSI-samples), according to the temperature and molar composition of the sample. Fluorine T_1_ experiments were carried out over a spectral width of 80 ppm for various delay time τ, ranging from 0.05–10 to 0.05–20 s (for BF_4_-samples) and from 0.05–10 to 0.05–40 s (for TFSI-samples), according to the temperature and molar composition of the sample. A total of eight transients per increment were collected for each T_1_ experiment for both ^1^H and ^19^F.

The spin–spin relaxation rates were measured using data matrices of 8192 (t_2_) × 16 (t_1_) and 32768 (t_2_) × 16 (t_1_) complex data points for ^1^H and ^19^F, respectively. Proton T_2_ experiments were carried out over a spectral width of 14 ppm for various echo time τ, ranging from 0.008–2 to 0.02–10 s (for BF_4_-samples) and from 0.008–2 to 0.02–15 s (for TFSI-samples), according to the temperature and molar composition of the sample. Fluorine T_2_ experiments were carried out over a spectral width of 80 ppm for various echo time τ, ranging from 0.008–2 to 0.04–10 s (for BF_4_-samples) and from 0.02–5 to 0.04–20 s (for TFSI-samples), according to the temperature and molar composition of the sample. A total of eight transients per increment were collected for each experiment T_2_ for both ^1^H and ^19^F.

The baselines of all arrayed T_1_ and T_2_ spectra were corrected prior to processing the data. Data were processed using an exponential filter in F_2_ dimension (with LB equal to 2 Hz for ^1^H and 0.5 Hz for ^19^F), and integrals were used in calculating relaxation times. Relaxation times were computed from experimental raw data by using the Bruker T_1_/T_2_ relaxation module with the standard one-component fitting function. Data were processed three times and errors were calculated from the maximum standard deviation found for the worst sample at the lowest temperature. Maximum errors are estimated to be 1% for T_1_ and 5% for T_2_.

Self-diffusion coefficients were measured by pulse gradient spin echo (PGSE) experiments by applying sine-shaped pulsed magnetic field gradients along the *z*-direction up to a maximum strength of *G* = 53.5 G cm^–1^. All the diffusion experiments were performed using the bipolar pulse longitudinal eddy current delay (BPP-LED) pulse sequence. Cation and anion self-diffusion coefficients were measured independently by carrying out PGSE experiments in the ^1^H and ^19^F frequency domains, respectively. All experiments were carried out over a spectral width of 14 ppm or 80 ppm for ^1^H and ^19^F, respectively, with a total of eight transients per increment. The relaxation delay was set to at least five times T_1_, and eight dummy scans were programmed prior to acquisition. The pulse gradients were incremented from 2 to 95% of the maximum gradient strength in a linear ramp with 32 steps. For each DOSY experiment, the duration of the magnetic field pulse gradients (δ) and the diffusion times (Δ) were optimized to obtain, where possible, 95% signal attenuation for the slowest diffusion species at the last step experiment. For ^1^H diffusion experiments, δ values were in the 1.6–6 ms range (for BF_4_-samples) and in the 2–6 ms range (for TFSI-samples), while Δ values were 0.6–0.8 s long (for both BF_4_- and TFSI-samples). For ^19^F diffusion experiments, δ values were in the 2.4–6 ms range (for BF_4_-samples) and in the 3–6 ms range (for TFSI-samples), while Δ values were 0.6–0.8 s long (for both BF_4_- and TFSI-samples). The baselines of all arrayed spectra were corrected prior to processing the data. Data were processed using an exponential filter in F_2_ dimension (LB = 0.5 Hz), and integrals were used in calculating relaxation times. The determination of self-diffusion coefficients used the Bruker T_1_/T_2_ module of TopSpin for each peak. The precision of the measured diffusion coefficient is estimated to be within 5%.

On equimolar mixtures (samples BF_4_-5:5 and TFSI-5:5), 2D NMR rotating frame nuclear Overhauser enhancement (ROESY) and heteronuclear Overhauser effect (HOESY) experiments were recorded at 305 K.

The ^1^H-^1^H ROESY experiments were performed by using the phase-sensitive off-resonance 2D ROESY pulse sequence (troesyph in the Bruker library) that minimizes the frequency offset effects. Spectra were recorded using eight transients over 8192 (t_2_) × 1024 (t_1_) complex data points. For both samples, 32 dummy scans and a mixing time of 150 ms under the spin lock conditions were used. The relaxation delay was set to 7.4 s for BF_4_-5:5 and 5 s for TFSI-5:5. The ROESY data sets were processed by applying a sine squared window function in both dimensions (SSB = 2) prior to the Fourier transform.

The ^1^H-^19^F HOESY experiments were acquired using the phase-sensitive echo-antiecho pulse sequence (hoesyetgp in the Bruker library). Spectra were recorded using four transients over 1024 (t_2_) × 128 (t_1_) complex data points. For both samples, 32 dummy scans and a 8 s long relaxation delay were used. For each sample, spectra were acquired at three mixing times (20, 50, and 100 ms). The HOESY data sets were processed by applying a sine squared window function in both dimensions (SSB = 2) and zero-filling to 2048 (t_2_) × 256 (t_1_) prior to the Fourier transform.

## Results and Discussion

Among all IL families, the most widely investigated, for both academic and application goals, have been those based on nonsymmetrically substituted *N*,*N′*-dialkylimidazolium cations, mainly 1-alkyl-3-methylimidazolium-based ILs.^39^ [BF_4_]^−^ is one of the most popular anions. In the series of 1-alkyl-3-methylimidazolium tetrafluoroborate salts, [C*_n_*mim][BF_4_], those with short alkyl chain lengths (*n* = 2–10) are liquids at room temperature, whereas the longer chain salts (*n* = 12–18) are low melting solids with an extensive thermotropic mesophase range.^40^ The sample BF_4_-10:0 displayed indeed a liquid crystalline phase at temperatures below 320 K. None of the BF_4_-mixtures showed mesophase transition in the temperature range used in this work. It is worthwhile to note that we also prepared a mixture with a large excess of [C_12_mim][BF_4_], with a molar ratio [C_12_mim]^+^:[C_2_mim]^+^ equal to 9.8:0.2, to explore the effect of [C_2_mim]^+^-doping on the liquid crystalline phase. Also in such sample, no stable thermotropic mesophase has been detected. Besides tetrafluoroborate, the anion bis(trifluoromethanesulfonyl)amide [TFSI]^−^ is largely used because it forms liquid salts of low viscosity with high thermal and electrochemical stability.^39^ In the series of 1-alkyl-3-methylimidazolium bis(trifluoromethylsulfonyl)imide salts, [C*_n_*mim][TFSI], no liquid crystalline phase has been detected.

To get a perspective on the structure and dynamics in the selected mixtures, multiple NMR experiments were performed on samples of Table 1. NMR parameters are clearly affected by the macroscopic properties of the samples, viscosity being probably the most important one. The change in viscosity in [C_12_mim][C_2_mim][TFSI] mixtures and [C_6_mim][C_2_mim][BF_4_] mixtures have been measured.^17,18,30^ A discussion on the deviation from ideality of the mixtures is out of the scope of the work, but for the interested readers, data from the literature and corresponding analysis in terms of mixing laws are reported in the Supporting Information. What is important for the present study is that, in all reported cases, the viscosity decreases with the increase in the molar fraction of the cation with the short alkyl chain (i.e., going from pure [C_12_mim][TFSI] to pure [C_2_mim][TFSI] or similarly from pure [C_6_mim][BF_4_] to pure [C_2_mim][BF_4_]). Even if, to the best of our knowledge, experimental data on [C_12_mim][C_2_mim][BF_4_] mixtures are not available, it is reasonable to assume that also in such system, the viscosity will decrease with increasing molar fraction of [C_2_mim]^+^. On the other hand, the macroscopic change in viscosity has been observed qualitatively during sample preparation and is also reflected in terms of linewidth in NMR spectra.

### NMR Dynamics

NMR relaxation and self-diffusion studies provide information on the dynamics of ILs and IL mixtures. NMR self-diffusion studies offer molecular level information on the translational motion, whereas the rotational information at the atomic level can be obtained from NMR relaxation studies.^36,37^

Relaxation times T_1_ and T_2_ and self-diffusion coefficients were measured for both the cation and the anion, using ^1^H and ^19^F NMR, respectively. Note that for the ionic liquid crystal [C_12_mim][BF_4_], the proton relaxation times and diffusion coefficients could be measured only in the isotropic range (325 and 320 K), while the fluorine values were obtained in the whole compositional range.

**Figure 2:**
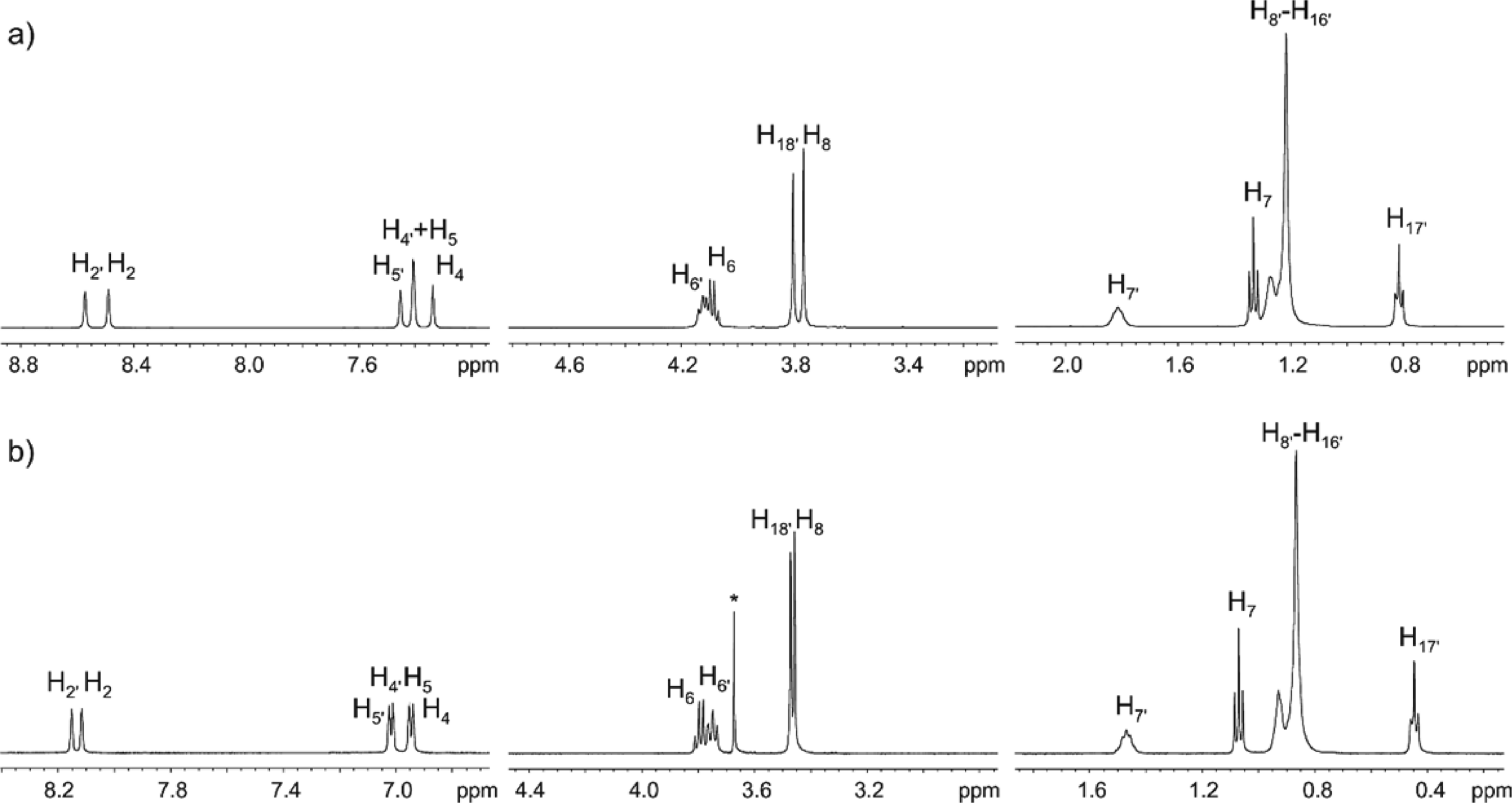
1D ^1^H NMR spectra at 325 K of (a) BF_4_-5:5 and (b) TFSI-5:5 (the peak labeled with an asterisk corresponds to an impurity in the capillary).

In the ^1^H spectra of the mixtures (Figure 2 and Figure S2), most peaks overlap, and hence, the corresponding relaxation times and diffusion coefficients are an average over the two cation species. The peaks at the lowest field corresponding to the H_2_ and H_2′_ protons and the peaks corresponding to H_7_ and H_17′_ protons of the terminal methyl groups of the cations (see Figure 1 for atom numbering) are relatively isolated and will be used in the following as representative peaks for each cation, [C_2_mim]^+^ or [C_12_mim]^+^, in the mixture.

In the ^19^F spectra, a single signal is observed for all the equivalent fluorine atoms in the anions. Note however that the fluorine signal of [BF_4_]^−^ shows two resonances due to the ^10^B/^11^B isotope effect.

### Rotational Dynamics

The spin–lattice (longitudinal, T_1_) and spin–spin (transverse, T_2_) relaxation times of nuclear spins are sensitive to both intra and intermolecular relaxation mechanisms and can be used to probe the rotational dynamics of the system. Several NMR active nuclei such as ^1^H, ^13^C, ^19^F, ^31^P, ^14^N, ^35^Cl, ^2^H, and ^7^Li have been used to characterize pure ILs as well as their mixtures with water and lithium salts.^41−54,^

**Figure 3:**
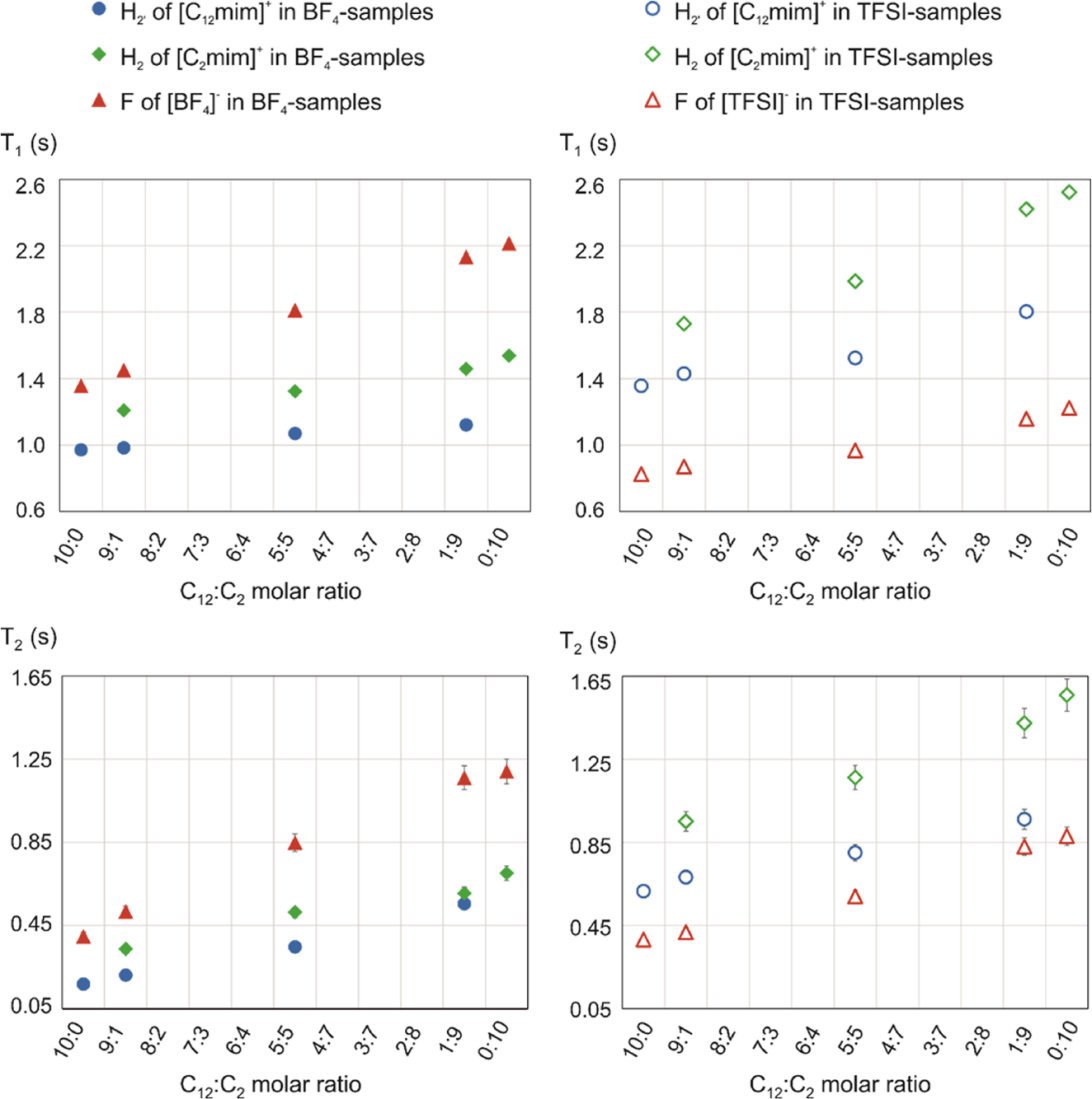
T_1_ and T_2_ relaxation times measured at 325 K for protons H_2_ and H_2′_ of the cations [C_12_mim]^+^ and [C_2_mim]^+^ and for fluorine of the anion [BF_4_]^−^ or [TFSI]^−^ in BF_4_-samples (on the left) and TFSI-samples (on the right). T_1_ and T_2_ are estimated to be accurate within ±1% and ±5%, respectively. When not visible, error bars are within the marker.

The temperature dependences of ^1^H and ^19^F T_1_ and T_2_ have been measured in the range 300–325 K. In all cases, both T_1_ and T_2_ increase with the temperature and no T_1_ minimum was found in the considered temperature range. The T_1_ and T_2_ relaxation times measured for the isolated H_2_ and H_2′_ protons of the cations and for the fluorine of the anions in all samples are reported for all temperatures in Tables S1–S3 and Figures S3 and S4 of the Supporting Information. As an example, Figure 3 shows the comparison between the T_1_ and T_2_ values found at 325 K in all samples as a function of the molar ratio of the two components. In all cases, T_1_ and T_2_ values increase with the increase in the molar fraction of [C_2_mim]^+^, which can be related to the decrease in viscosity of the sample. Within each series (with [BF_4_]^−^ or [TFSI]^−^ as the anion), the T_1_ and T_2_ values of proton H_2_ of [C_2_mim]^+^ are bigger than those of proton H_2′_ of [C_12_mim]^+^. This is due to the size of the cations, with the smaller one ([C_2_mim]^+^) showing a faster rotational motion than the bigger one ([C_12_mim]^+^). Comparing the two series, T_1_ and T_2_ values of the H_2_ and H_2′_ protons of both cations are bigger when [TFSI]^−^ is present as the counterion. To a first approximation, this can be related to the nature of the anion, as [TFSI]^−^ is well known for its promotion of fluidity,^16^ and gives in general less structured materials compared to [BF_4_]^−^.

A particularly informative parameter is the T_1_/T_2_ ratio as it can be used as a very sensitive probe of local order in the system.^55,56^ For a common isotropic mixture, the T_1_/T_2_ ratio is equal to 1. Intermediate values close to one are typical of viscous liquids, and higher values indicate the presence of local structures in the system.^55,56^ T_1_/T_2_ ratios calculated for the selected H_2_ protons of the cations and for the fluorine of the anions in all samples are reported for all temperatures in Tables S1–S3 and Figure S5 of the Supporting Information.

**Figure 4:**
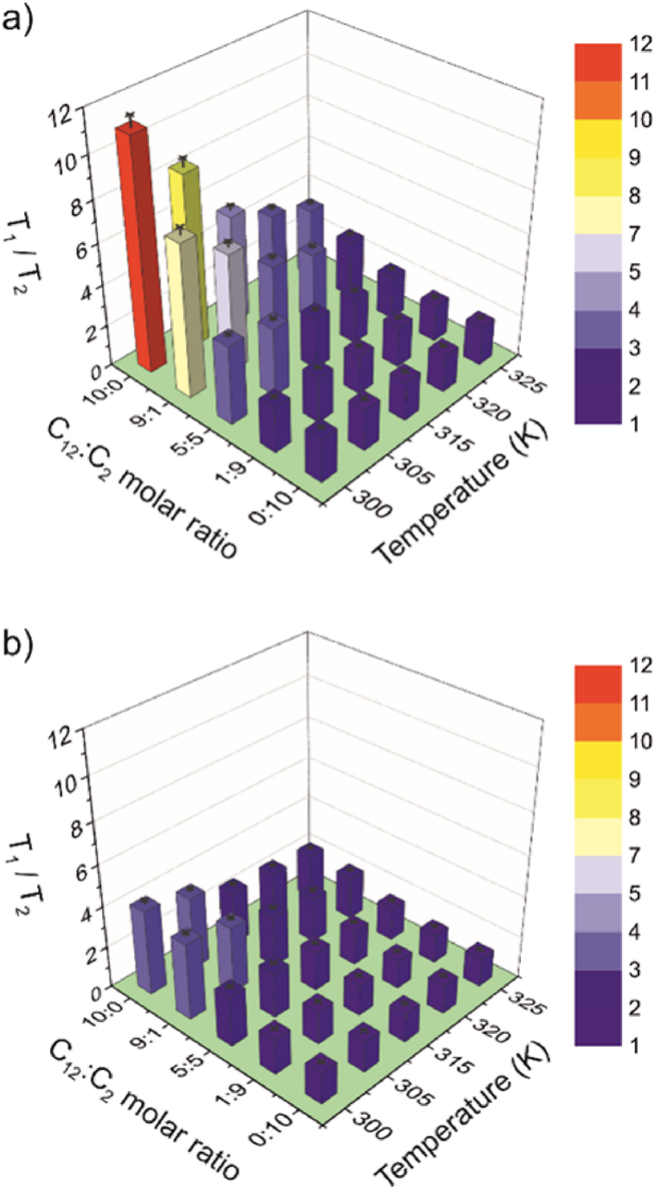
T_1_/T_2_ ratios calculated for the fluorine of the anions (a) [BF_4_]^−^ and (b) [TFSI]^−^ in all samples. T_1_/T_2_ are estimated to be accurate within ±5%.

Figure 4 reports the T_1_/T_2_ ratios calculated for the fluorine of the anions in all samples at all experimental temperatures. It can be observed that in both cases, the T_1_/T_2_ ratio progressively decreases with increasing temperature and increasing molar fraction of the smaller cation ([C_2_mim]^+^). The maximum T_1_/T_2_ value found when [TFSI]^−^ is the counterion (at 300 K and for a C_12_:C_2_ molar ratio of 10:0) is 4.1, whereas it is 11.1 for [BF_4_]^−^. This is a confirmation of the significantly more ordered nanostructure formed with tetrafluoroborate. It has been reported for different ILs that T_1_/T_2_ ratios between 1.0 and 2.6 are not sufficiently high to confirm the presence of sustained local order on a timescale of the inverse Larmor frequency of the nucleus under study.^56,57^ In the samples studied in this work, we can observe that the TFSI-mixtures behave overall as an isotropic medium in the whole compositional range on the timescale of ∼1/ω, with T_1_/T_2_ ratios between 1.4 to 4.4. On the contrary, T_1_/T_2_ ratios found for BF_4_-mixtures point toward the existence of local structures formed on the timescale of ∼1/ω in the samples with a significant amount of [C_12_mim]^+^ (BF_4_-10:0, BF_4_-1:9, and BF_4_-5:5) and at the lowest temperatures.

For comparison, the T_1_/T_2_ ratios calculated for the H_2_ and H_2′_ protons of the cations in all samples at all experimental temperatures are reported in Figure S6.

From temperature-dependent T_1_ data, it is possible in principle to calculate the activation energy of the rotational motion at a given site when ω_0_τ_C_ ≪ 1 (see Supporting Information for the theoretical background).^44^ It should be noted that ionic liquids may show strong deviations from Arrhenius behavior when relaxation measurements over wide temperature ranges are performed.^58^ However, in the present work, a quite narrow temperature range has been considered and to a first approximation, the temperature-dependent correlation time τ_C_ obeys the Arrhenius equation (Figures S7 and S8). It is thus possible to estimate the activation energy of reorientational motion in the studied temperature interval.^44^ An additional noteworthy point here is that structurally different protons may exhibit different T_1_ values as the result of characteristic intramolecular dynamics of the given segment, which contribute to the relaxation, in addition to the overall reorientation of the cation.^58^ It follows that one can calculate an “apparent” activation energy averaged over several types of movements.^47^ The apparent activation energy of the rotational motion, *E*_a_^rot^, estimated from the linear dependence of T_1_ relaxation times in the range 300–325 K for protons H_2_ and H_7_ of [C_2_mim]^+^, protons H_2′_ and H_17′_ of [C_12_mim]^+^, and ^19^F of [BF_4_]^−^ and [TFSI]^−^ are given in Table 2 and Figure S9. Note that in the BF_4_-mixtures, the signal corresponding to proton H_7_ is close to the intense signal from the methylene protons of the long alkyl chain of [C_12_mim]^+^, so the integration of the isolated peak was not possible for BF_4_-9:1.

**Table 2:** Apparent Activation Energies *E*_a_^rot^ (kJ mol^–1^) Obtained from Temperature-Dependent T_1_ Relaxation Times in the Range 300–325 K at Given Proton and Fluorine Sites for the Pure ILs and their Mixtures^a^

	BF_4_-mixtures				
C_12_:C_2_ molar ratio	H_2′_ of [C_12_mim]^+^	H_2_ of [C_2_mim]^+^	H_17′_ of [C_12_mim]^+^	H_7_ of [C_2_mim]^+^	[BF_4_]^−^
10:0					b
9:1	6.9	7.1	16.3	b	8.8
5:5	6.2	5.5	13.7	11.5	9.2
1:9	5.3	8.1	13.6	13.6	8.2
0:10		9.3		14.1	7.9

	TFSI-mixtures				
C_12_:C_2_ molar ratio	H_2′_ of [C_12_mim]^+^	H_2_ of [C_2_mim]^+^	H_17′_ of [C_12_mim]^+^	H_7_ of [C_2_mim]^+^	[TFSI]^−^
10:0	11.7		13.9		9.1
9:1	8.8	9.6	13.6	11.3	11.6
5:5	7.7	9.4	13.0	13.8	12.7
1:9	8.5	10.4	13.3	14.2	13.9
0:10		11.9		23.8	14.5

a Values are estimated to be accurate within ±5%.

b Values not available

Note that local reorientation processes affect to some extent the relaxation data of the selected protons,^47,58^ and the same holds also for ^19^F relaxation data, largely governed by CF_3_ and BF_4_ reorientations. Therefore, the results should be interpreted bearing in mind that structural relaxation and intramolecular motion overlap but can anyway give some insights into segmental reorientation. Moreover, from the T_1_/T_2_ ratio, we can assume that in the TFSI-mixtures, the extreme narrowing approximation can be applied, but the BF_4_-mixtures might locate more in the intermediate region, at least for some compositional ranges, then the *E*_a_^rot^ may be biased.^46^ However, the order of magnitude found here for the activation energies are in line with data from the literature. For instance, for [C_1_mim][TFSI], the *E*_a_^rot^ estimated from the linear part of the proton T_1_ curve (from 233 to 293 K) at low fields in the crystalline state was approximately 8.7 kJ mol^–1^ and was associated with the rotational motion of the methyl group.^43^ An *E*_a_^rot^ of 12.5–15.1 kJ mol^–1^ was also found for the crystalline states of [C_4_mim][PF_6_] and assigned to the δ-CH_3_ rotation.^59^ In ILs composed of *N*-methyl-*N*-propyl-pyrrolidinium (P_13_) with two anions, TFSI and FSI, the three-type protons have similar activation energies (ca. 15 kJ mol^–1^ for FSI-systems and 17 kJ mol^–1^ for TFSI systems).^60^*E*_a_^rot^ for alkyl protons of [C_4_mim][BF_4_] calculated from temperature-dependent spin–lattice relaxation rate at the different sites were in the range 15–19 kJ mol^–1^,^47,61^ and *E*_a_ for H_7_ of [C_2_mim][BF_4_] was 16.1 kJ mol^–1^.^61^ As for the fluorine, the activation energy calculated from ^19^F T_1_ values was ca. 14 kJ mol^–1^ (above 303 K) in [P_13_][TFSI], 14.1 kJ mol^–1^ (above 283 K) in [C_2_mim][TFSI], 11.5 kJ mol^–1^ (above 288 K) in [C_4_mim][BF_4_], and 10.8 kJ mol^–1^ (above 303 K) in [C_2_mim][BF_4_].^54,60,61^

Therefore, while bearing in mind the restriction of the obtained apparent activation energies in the considered temperature range, a number of considerations can be drawn from Table 2:

- *E*_a_^rot^ for [BF_4_]^−^ (7.9–9.2 kJ mol^–1^) are smaller than *E*_a_^rot^ for [TFSI]^−^ (9.1–14.5 kJ mol^–1^). This suggests that the rotational motion can be activated more easily for [BF_4_]^−^ than for [TFSI]^−^ due to the smaller ion size and the tetrahedral symmetrical structure.^61^
- *E*_a_^rot^ for H_17′_ in both BF_4_- and TFSI-mixtures slightly decrease with increasing [C_2_mim]^+^ molar fraction, and in TFSI-mixtures, it is almost independent on the composition of the mixture (13.9–13.0 kJ mol^–1^). This can be explained considering that the terminal methyl group of the long chains feels always a very similar environment, that is the apolar chains of the neighboring cations. In other words, even if the progressive increase of the smaller cation affects the whole nanostructure of the system, the inner core of the polar domains does not change dramatically. On the contrary, *E*_a_^rot^ for H_7_ increases, more markedly in TFSI-mixtures (11.3–23.8 kJ mol^–1^). This is because the small cations are located mostly in the polar domain, which suffers more significantly from the change in the molar ratio between the smaller and the bigger cation.
- A clear increase in *E*_a_^rot^ is also seen for ^19^F in TFSI-mixtures (9.1–14.5 kJ mol^–1^), in line with the trend for H_7_. Contrarily, *E*_a_^rot^ for ^19^F in BF_4_ mixtures is almost constant.

The latter point is likely the most striking finding. If interpreted together with results for the two cations, the different behavior displayed by the two anions would indicate that [BF_4_]^−^ is located steadily in the polar domain, whose inner composition and arrangement do not change significantly with the change in the molar ratio of the two components. This would explain why the activation energy required for a substantially local motion does not vary and confirms the role of [BF_4_]^−^ as a strong counterion. The scenario is different for the more conformationally flexible anion with extensive charge delocalization, [TFSI]^−^, which probe both polar and apolar domains and is then sensitive to the nanostructural modifications that occur upon dilution of [C_12_mim][TFSI] with [C_2_mim][TFSI]. The effect of “encroachment” has been already observed for anions such as [TFSI]^−^ and related both to the size, which exceeds that of the imidazolium heads, and to the low basicity, which reduces the strength of the polar interactions.^13^ Overall, this translates into a weakly interacting nature of the anion that easily oversteps the bounds of the polar domains toward the apolar chains. Even considering that the proposed T_1_ analysis produces apparent *E*_a_^rot^ values in a narrow temperature range, the results for the two series reveal a substantially different behavior of the two anions and could be read as a dynamic fingerprint of the short-range mobility in IL mixtures.

### Translational Dynamics

Pulsed field gradient (PFG) NMR has been largely used to measure diffusion in several ILs and IL mixtures.^9,11,16,38,51,55,60,62−71^ The self-diffusion coefficients *D* of the anions and cations in all the samples were measured independently by PFG experiments in the ^19^F and ^1^H frequency domains, respectively. The resulting self-diffusion coefficients are reported for all temperatures in Tables S5–S7 and Figure S10 of the Supporting Information.

**Figure 5:**
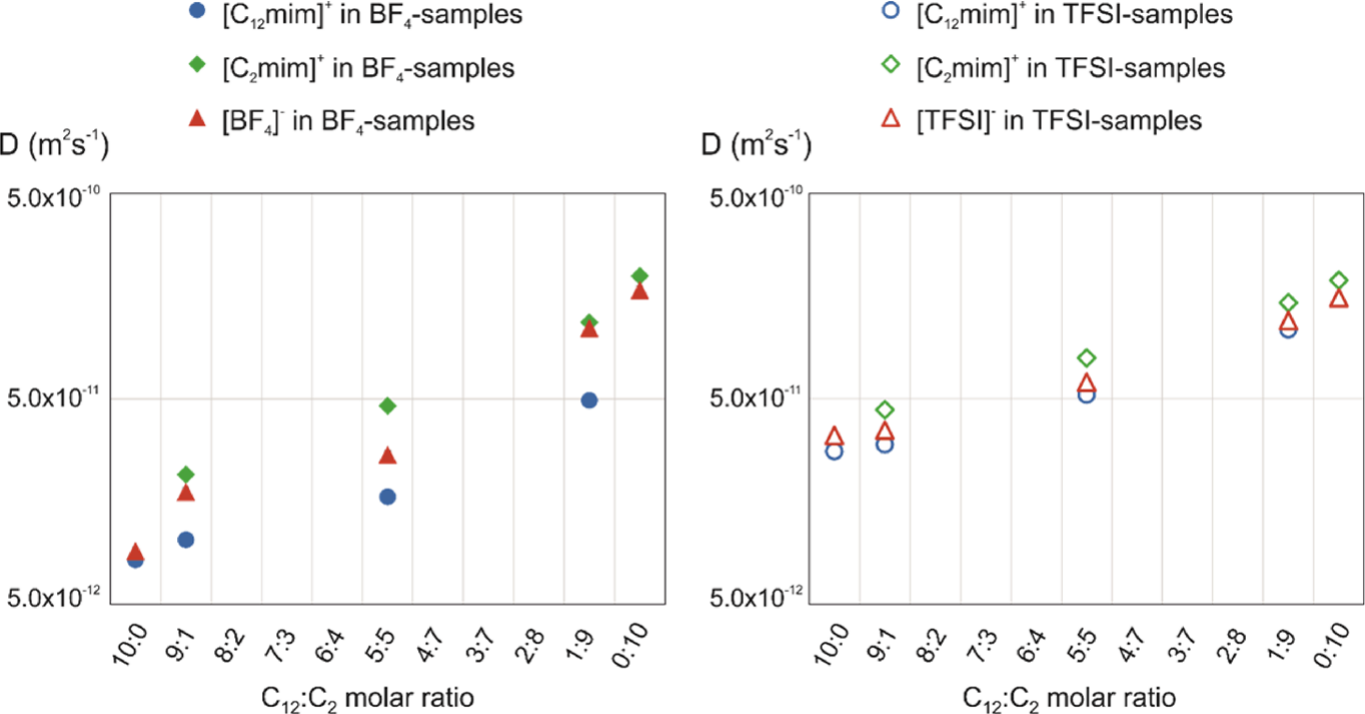
Diffusion coefficients measured at 325 K for the cations [C_12_mim]^+^ and [C_2_mim]^+^ and the anion [BF_4_]^−^ or [TFSI]^−^ in all samples. *D* are estimated to be accurate within ±5%. When not visible, error bars are within the marker.

Figure 5 shows as an example the comparison between the diffusion coefficients found at 325 K for the cations [C_12_mim]^+^ and [C_2_mim]^+^ and the anions [BF_4_]^−^ and [TFSI]^−^ in all samples as a function of the molar ratio of the two components of the mixtures. In all cases, the mobility increases with the increase in the molar fraction of the cation with the shorter chain ([C_2_mim]^+^) in the mixture. Again, this observation can be explained by the decrease in the viscosity of the mixture, as also observed in other IL mixtures.^14^

As previously found,^66^ in neat ILs, the self-diffusion coefficients of cations are faster than those of the anion in the [C_2_mim]^+^-samples and slower in the [C_12_mim]^+^-samples. For the series of [C*_n_*mim][TFSI] ILs, it has been reported that the self-diffusion coefficients of cations are faster as compared to the anion in shorter alkyl group substitution ILs and slightly slower in longer alkyl group substitution ILs, with a crossover in hexyl or octyl (*n* = 6–8) group substitution.^58,66^ Here, the system is complicated by the simultaneous presence of two cations, one with a short ethyl chain and the other with a long dodecyl chain. In all mixtures, the self-diffusion coefficients of the anion turned out to be an intermediate value between those of the two cations.

Comparing the two series, in the neat ILs with the longer chain ([C_12_mim][BF_4_] and [C_12_mim][TFSI]) and in all mixtures (9:1, 5:5, and 1:9), both cations diffuse faster when [TFSI]^−^ is present as the counterion with respect to [BF_4_]^−^. To a first approximation, this can be related to the nature of the anion as [TFSI]^−^ gives in general more fluid and less structured materials compared to [BF_4_]^−^. As for the anions, it can been observed that [TFSI]^−^ diffuses faster than [BF_4_]^−^ in the neat ILs with the longer chain ([C_12_mim][BF_4_] and [C_12_mim][TFSI]) and in the mixtures with C_12_:C_2_ molar ratios equal to 9:1 and 5:5. Both anions have similar diffusion coefficients in the neat ILs with the shorter chain ([C_2_mim][BF_4_] and [C_2_mim][TFSI]) and in the mixture with a C_12_:C_2_ molar ratio equal to 1:9.

The dependence of the transport properties of the mixtures against temperature was also investigated. A linear Arrhenius temperature behavior has been observed for the diffusion coefficients of all samples over the 300–325 K temperature range (Figure S11). Note again that the Arrhenius behavior depends strongly on the location of the considered temperature interval with respect to the transition temperatures of the material, and strong deviations from the Arrhenius law have been described for neat [C*_n_*mim][TFSI] ILs over wide temperature ranges.^58^ As expected, the diffusion coefficient for the anion in the neat ionic liquid crystal [C_12_mim][BF_4_] does not change linearly with the temperature over the 300–325 K range, but a somehow linear trend can be observed in the anisotropic range 315–300 K. The apparent activation energies calculated from ion diffusion measurements are listed in Table 3 and displayed in Figure S12 as a function of the molar ratio of the two components of the mixtures (see Supporting Information for the theoretical background).

**Table 3:** Apparent Activation Energies *E*_a_^transl^ (kJ mol^–1^) Obtained from Temperature-Dependent Diffusion Data for the Pure ILs and their Mixtures^a^

	BF_4_-mixtures			TFSI-mixtures		
C_12_:C_2_ molar ratio	[C_12_mim]^+^	[C_2_mim]^+^	[BF_4_]^−^	[C_12_mim]^+^	[C_2_mim]^+^	[TFSI]^−^
10:0			49.3^b^	43.5		44.1
9:1	51.7	55.4	51.7	41.9	40.5	41.3
5:5	42.1	38.2	39.0	41.5	37.0	40.7
1:9	44.0	32.3	35.8	44.3	34.8	44.7
0:10		40.6	42.3		37.3	46.6

a Values are estimated to be accurate within ±5%.

b Calculated only in the anisotropic range.

In all cases, the activation energy is not linear but shows a minimum value at a given composition. The change in the apparent activation energy in the studied compositional range is greater for BF_4_-mixtures than for TFSI-mixtures. However, the trends found for the two cations are quite similar in the two series. The smaller cation [C_2_mim]^+^ shows the lowest *E*_a_^transl^ for the samples BF_4_-1:9 and TFSI-1:9, while the minimum is observed for the equimolar mixtures (BF_4_-5:5 and TFSI-5:5) in the case of the bigger cation [C_12_mim]^+^. Next to the viscosity effect, which decreases going from pure [C_12_mim][X] to pure [C_2_mim][X], to rationalize these trends, one could consider also the “disturbing effect” exerted by the addition of a cation with different size to a pure IL. This means that when a 10 mol % of [C_12_mim][X] is added to pure [C_2_mim][X], the structure of the IL is disturbed so that the small cation moves slightly more easily in the 1:9 mixture. Vice versa, if one considers the addition of a small cation to pure [C_12_mim][X], the effect is not that strong until the molar fraction of [C_2_mim]^+^ gets significant.

Comparing the *E*_a_^transl^ of the two anions in the two series, a markedly different behavior emerges again. In BF_4_-mixtures, the diffusive motion of the anion appears to be strongly correlated with that of the smaller cation in all mixtures but the sample with a higher molar ratio of [C_12_mim]^+^ (BF_4_-9:1), where [BF_4_]^−^ and [C_12_mim]^+^ have the same activation energy. In TFSI-mixtures, the anion [TFSI]^−^ and the cation with the longer chain ([C_12_mim]^+^) have very similar activation energies in the whole compositional range, with slight changes and a minimum value for the sample TFSI-5:5. The diffusive motion of the smaller cation [C_2_mim]^+^ appears to be less correlated and its activation energy is always smaller. The *E*_a_^transl^ of the anion can be seen then as an important dynamic probe in IL mixtures: the larger and more diffuse [TFSI]^−^ explores both polar and apolar domains and its diffusive motion follows more tightly that of the amphiphilic cation, while the smaller and charge localized [BF_4_]^−^ moves rather within the polar regions so that its diffusive motion is closer to that of [C_2_mim]^+^.

Finally, it should be noted that in the considered temperature interval, the apparent activation energies associated with relaxation data are of 5–24 kJ mol^–1^ (see Table 2) and the apparent activation energies associated with diffusion data are larger (32–52 kJ mol^–1^) (see Table 3). Such a difference is not surprising if one considers the molecular motion that affects these apparent activation energies, rotational versus translational, and is in agreement with values found for other ILs. For instance, in P_13_-FSI and P_13_-TFSI, the activation energies were 15–18 kJ mol^–1^ for the rotational motion and much larger for translational diffusion (25 kJ mol^–1^ for P_13_-FSA and 30 kJ mol^–1^ for P_13_-TFSA).^60^ Indeed, it should be remembered again that what dominates the observed T_1_ data is not the correlation time of the reorientation of the ion as a whole, but the characteristic time for the rotation of a segment. In this sense, the T_1_ and *D* analyses can be seen as complementary tools to probe the dynamics of the system at different scales.

### NMR Structure

Valuable information for the assessment of the local structure of ionic liquids, including ion–ion interactions, can be obtained from the intermolecular nuclear Overhauser enhancement (NOE).^72^ The NOE originates from dipolar cross-relaxation between nuclear pairs and thus gives information on the proximity between the molecular sites involved in the interactions. In the investigated mixtures, relative proximities for cation–cation and cation–anion interactions were studied separately by means of two 2D experiments: {^1^H-^1^H} rotating frame NOE correlation (ROESY) and {^1^H-^19^F} heteronuclear NOE correlation (HOESY) experiments as they contain different nuclides with *I* = 1/2.

The interpretation of the NOE data for ILs showed a rapid evolution from the atom-pair interpretation based on short-distance interactions^72−76^ to a generalized model including also long-range effects, first introduced by Weingärtner in 2013.^77^ The authors demonstrated that intermolecular NOEs were influenced by the Larmor frequency of the interacting nuclei, with the important fallout that the intermolecular NOEs of nuclei with similar Larmor frequency, such as in {^1^H-^1^H} or {^1^H-^19^F} NOE experiments, explore not only the first neighbor contacts but also long-range interactions. Additionally, different HOESY experiments are expected to provide different types of distance-dependent information on the interacting atoms as a function of their relative Larmor frequencies. For example, in Li^+^-doped ILs with fluorine containing anions, typically employed for electrochemical applications, {^1^H-^1^H} and {^1^H-^19^F} contain information on interaction distances beyond the conventional threshold of 4 Å for vanishing NOE, while {^1^H-^7^Li} NOEs are largely dominated by short-range interactions.^78,79^

#### ^1^H-^1^H Proximity: Homonuclear Experiments (ROESY)

^1^H-^1^H ROESY is one of the 2D NMR methods for correlating signals arising from protons close in space.^80^ The ROESY correlation peaks are the result of cross-relaxation between neighboring protons, with the main mechanism being the through-space dipole–dipole interaction. The cross-peak intensity reflects the extent of magnetization transfer between interacting nuclei and, in the case of intramolecular NOE, i.e., in the case of fixed internuclear distances, it is inversely proportional to the sixth power of their internuclear distance. According to the Weingärtner model, the intermolecular NOE are frequency-dependent and also affected by time-dependent internuclear distances. In other words, the internuclear distances are modulated by the rotational and translational dynamic properties of the ions, leading to a distance dependence of NOE no longer scaling as r^–6^ but rather as r^–*n*^ with typically 1 < *n* < 6. The quantitative extraction of intermolecular distances via intermolecular NOEs is not the focus of this work and it is thoroughly described by Martin et al.^81^ As a consequence of the r^–*n*^ distance scaling of the intermolecular NOEs, their intensity spots on spins far beyond the first coordination layer. This would mean that site-specific NOE measurements reflect the mean orientation of the ions over long distances rather than the local structure of distinct ion aggregates.

In the present work, we used ^1^H-^1^H NOE data to provide details on the intermolecular cation–cation organization in the studied mixtures by a qualitative evaluation of the cross-peaks among the different protons of the two components.

Figure S13 shows the ROESY spectrum recorded at 305 K for the sample BF_4_-5:5. The pattern is overall similar to that found for neat [C*_n_*mim]-based ILs with *n* = 6, 8, and in general, the ROESY cross-peaks evidence an IL molecular organization close to that already proposed for other neat 1-alkyl-3-methylimidazolium salts with aromatic ring associations and possible head-to-tail and tail-to-tail contacts.^73,82^

From the standpoint of the IL mixture, it is interesting to find evidence of intermolecular correlations between the two types of cations. Many proton signals are overlapped in ^1^H NMR spectra of BF_4_- and TFSI-mixtures, making the assignment of many cross-peaks ambiguous. Luckily, in the aromatic part, peaks are not fully overlapping, and cross-peaks in this area can give hints on the nanostructural organization of the mixture. Indeed, the cross-peaks between H_2_ of [C_2_mim]^+^ and H_5′_ of [C_12_mim]^+^ and between H_2′_ of [C_12_mim]^+^ and H_4_ of [C_2_mim]^+^ reasonably suggest the presence of intermolecular interactions between the two different cations and thus imply the existence of mixed ring assemblies (see inset in Figure S13). Note that due to the overlap of signals corresponding to H_5_ of [C_2_mim]^+^ and H_4′_ of [C_12_mim]^+^, we would rather avoid any interpretation of the corresponding cross-peaks. The main conclusion emerging from the observed ^1^H-^1^H NOEs in the equimolar BF_4_-5:5 mixture is that the cations’ heads show significant short contacts despite the repulsive Coulombic interaction, in line with the existence of polar domains in the mixture.

The ROESY spectrum recorded at 305 K for the sample TFSI-5:5 is somehow different (Figure S14). ROESY cross-peaks are detectable between H_2_ and H_4_ and H_2_ and H_5_ of [C_2_mim]^+^ or between H_2′_ and H_5′_ of [C_12_mim]^+^ (see inset in Figure S14). Such ring intermolecular interactions between the same cations are compatible with the formation of polar domains. This observation is substantiated by the presence of cross-peaks between H_2_ and H_6_, H_2_ and H_8_, H_4_ and H_8_, and H_5_ and H_6_ of [C_2_mim]^+^, or similarly between H_2′_ and H_18′_, H_2′_ and H_6′_, H_4′_ and H_18′_, and H_5′_ and H_6′_ of [C_12_mim]^+^ (see inset in Figure S14). No clear ROESY peaks between signals of the two different cations are observed here.

The overall picture seems to indicate a quite different situation in the two equimolar mixtures, with the BF_4_-sample showing clean ROESY peaks between the ring’s protons of both the same and different cation species, and the TFSI-mixture providing evidence of some interactions between the same cation species but no clear intermolecular contacts between [C_2_mim]^+^ and [C_12_mim]^+^.

In summary, the presence or absence of intermolecular NOE interactions between the two types of cations in BF_4_- or TFSI-mixture, respectively, might point toward a different nanostructure in the two samples. In the BF_4_-mixture, the small [C_2_mim]^+^ cation would be well intercalated between the [C_12_mim]^+^ heads, resulting in an intimate and “well-mixed” ring assemblies. In the TFSI-mixture, interactions are in general looser because of the anion’s nature, and well-ordered [C_12_mim]^+^-[C_12_mim]^+^ assemblies are formed, while the small cation [C_2_mim]^+^ would be probably too mobile to give detectable interactions with the [C_12_mim]^+^.

#### ^1^H-^19^F Proximity: Heteronuclear Experiments (HOESY)

This section reports on heteronuclear {^1^H-^19^F} 2D NOE spectroscopy (HOESY) to investigate anion–cation interactions by exploiting the fact that cations contain ^1^H but not ^19^F nuclei and vice versa for the anions.

**Figure 6:**
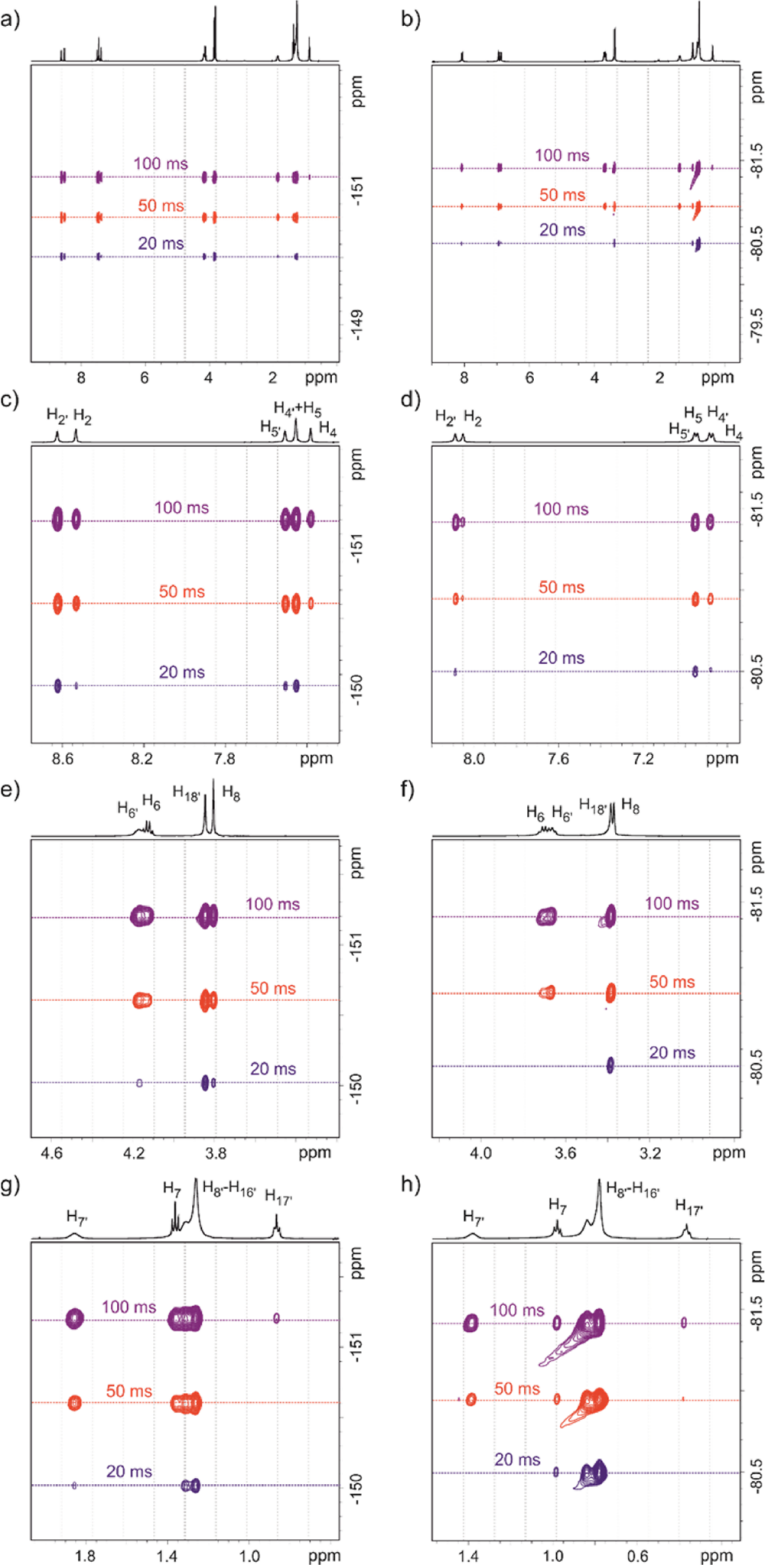
HOESY spectra at different mixing times (20, 50, or 100 ms) recorded at 305 K for (a) BF_4_-5:5 and (b) TFSI-5:5 and (c–h) enlargements of the different spectral regions.

**Figure 7:**
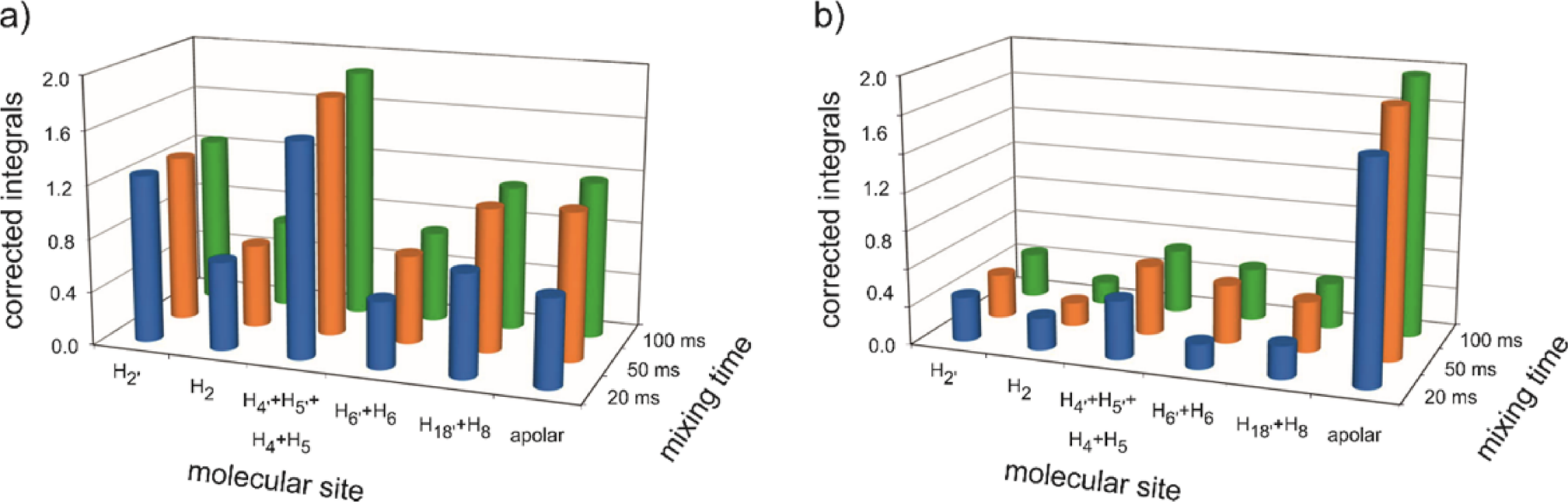
Corrected integrated peak volume of ^1^H-^19^F NOE cross-peaks at 20, 50, and 100 ms mixing time for (a) sample BF_4_-5:5 and (b) sample TFSI-5:5. See text for a description of the labels.

Figure 6 shows the HOESY spectra of the two equimolar mixtures BF_4_-5:5 and TFSI-5:5 acquired at three different mixing times. The cross signals between the protons of the cations and the fluorine nuclei of the anions are recognizable. All cation peaks of IL are interacting with the anion. The individual signal intensities give an idea of the order of magnitude of the particular cross relaxation and thus of the intensity of interaction. Even if a quantitative analysis of HOESY cross-peaks is out of the scope of the present work, to qualitatively interpret the HOESY spectra, the cross-peaks were integrated and the integrated HOESY intensities corrected by a factor *n*_H_*n*_F_/(*n*_H_ + *n*_F_), with *n*_H_ and *n*_F_, the number of ^1^H and ^19^F nuclei contributing to the observed NOE signal.^63,83,84^ Original and corrected cross-peak volumes are reported in Tables S8 and S9 and Figure 7. In particular, the histograms of Figure 7 are the result of the grouping of NOE contribution from different molecular sites. The histogram bars labeled as H_2_, H_2′_, H_4_, H_4′_, H_5_, H_5′_, H_6_, H_6′_, H_8,_ and H_18′_ correspond to the NOE between the fluorine nuclei of the anion and the protons on the polar domain of the ILs. The bar on the right hand corner of the plot is labeled as “apolar” and accounts for the cumulative NOE intensity between the fluorine nuclei of anion and all of the protons belonging to the alkyl chain of the cation and not contributing to the polar domain. In this way, the histograms offer a clear picture of the proximity of the anion to both domains of the ILs for both [BF_4_]^−^ and [TFSI]^−^. As expected, in sample BF_4_-5:5, the strongest interaction is with the head group and only weak interactions can be observed with the alkyl chain. The situation is different in TFSI-5:5, where the most significant interactions are observed between the anion and the alkyl chain, similar to what reported for [C_8_mim][TFSI].^83^

## Conclusions

We showed how a joint use of different NMR-derived descriptors, T_1_/T_2_ ratios, activation energy for T_1_ and for diffusion, and homo and heteronuclear intermolecular NOEs, contributes to a description of the polar and apolar domains in representative IL mixtures, underlining the role of the anion in dictating the dynamic features of the mixtures. The anions used in this study are paradigmatic of opposite classes: small, symmetric, charge-localized in one case ([BF_4_]^−^), and large, with conformation degrees of freedom, diffuse and polarizable charge in the other ([TFSI]^−^). The results above discussed coherently describe two different solvation modes of the anions: [BF_4_]^−^ dominated by Coulombic interactions with the polar heads (quite irrespective of the average alkyl chain length in the mixtures) and [TFSI]^−^ sensitive to a complex balance of Coulombic and dispersive interactions that allows extensive interaction with the palisade of the alkyl chains aggregation typical of the apolar domains. On the other way round, the relaxation and diffusion data can be used to validate the structural interpretation provided by the NOE data without passing through the rigorous but time-consuming protocol of full simulation of the build-up curves leading to the assessment of nuclei-pair distances. This point is quite important in view of a rethinking of the intermolecular NOE interpretation considering also the long-range effects. Finally, and from a methodological standpoint, the structural features of the anions remarked above, and their response to the NMR descriptors used in this work, indicate that those anions can be exploited as “dynamic probes” for gaining information of the polar and nonpolar regions of ionic liquids and their mixtures.

## Supporting Information Available

Brief survey of reported viscosity data; ^1^H and ROESY spectra; tables and plots reporting experimental T_1_, T_2_, T_1_/T_2_, and *D* values; Arrhenius plots and apparent activation energies obtained from temperature-dependent T_1_ and D data; tables reporting integrals from HOESY spectra; and theoretical background for the calculation of activation energy from relaxation and diffusion data (PDF)

## Abbreviations

BPP-LED: bipolar pulse longitudinal eddy current delay
CPMG: Carr–Purcell-Meiboom–Gill
DMSO: dimethyl sulfoxide
DOSY: diffusion ordered spectroscopy
HOESY: heteronuclear Overhauser enhancement spectroscopy
ILs: ionic liquids
IR: inversion recovery
NMR: nuclear magnetic resonance
NOE: nuclear Overhauser enhancement
PFG: pulsed field gradient
PGSE: pulse gradient spin echo
ROESY: rotating frame nuclear Overhauser enhancement.

## References

[ref1] Niedermeyer H.; Hallett J. P.; Villar-Garcia I. J.; Hunt P. A.; Welton T. Mixtures of Ionic Liquids. Chem. Soc. Rev. 2012, 41, 7780–7802. 10.1039/c2cs35177c. 22890419

[ref2] Welton T. Ionic Liquids: A Brief History. Biophys. Rev. 2018, 10, 691–706. 10.1007/s12551-018-0419-2. 29700779 PMC5988633

[ref3] Cabry C. P.; D’Andrea L.; Shimizu K.; Grillo I.; Li P.; Rogers S.; Bruce D. W.; Canongia Lopes J. N.; Slattery J. M. Exploring the Bulk-Phase Structure of Ionic Liquid Mixtures Using Small-Angle Neutron Scattering. Faraday Discuss. 2018, 206, 265–289. 10.1039/C7FD00167C. 28948252

[ref4] Hallett J. P.; Welton T. Room-Temperature Ionic Liquids: Solvents for Synthesis and Catalysis. 2. Chem. Rev. 2011, 111, 3508–3576. 10.1021/cr1003248. 21469639

[ref5] Plechkova N. V.; Seddon K. R. Applications of Ionic Liquids in the Chemical Industry. Chem. Soc. Rev. 2008, 37, 123–150. 10.1039/B006677J. 18197338

[ref6] Watanabe M.; Thomas M. L.; Zhang S.; Ueno K.; Yasuda T.; Dokko K. Application of Ionic Liquids to Energy Storage and Conversion Materials and Devices. Chem. Rev. 2017, 117, 7190–7239. 10.1021/acs.chemrev.6b00504. 28084733

[ref7] Chatel G.; Pereira J. F. B.; Debbeti V.; Wang H.; Rogers R. D. Mixing Ionic Liquids-″simple Mixtures″ or “Double Salts”?. Green Chem. 2014, 16, 2051–2083. 10.1039/c3gc41389f.

[ref8] Clough M. T.; Crick C. R.; Gräsvik J.; Hunt P. A.; Niedermeyer H.; Welton T.; Whitaker O. P. A Physicochemical Investigation of Ionic Liquid Mixtures. Chem. Sci. 2015, 6, 1101–1114. 10.1039/C4SC02931C. 29560198 PMC5811077

[ref9] Miran M. S.; Yasuda T.; Susan M. A. B. H.; Dokko K.; Watanabe M. Binary Protic Ionic Liquid Mixtures as a Proton Conductor: High Fuel Cell Reaction Activity and Facile Proton Transport. J. Phys. Chem. C 2014, 118, 27631–27639. 10.1021/jp506957y.

[ref10] Weber C. C.; Brooks N. J.; Castiglione F.; Mauri M.; Simonutti R.; Mele A.; Welton T. On the Structural Origin of Free Volume in 1-Alkyl-3-Methylimidazolium Ionic Liquid Mixtures: A SAXS and ^129^Xe NMR Study. Phys. Chem. Chem. Phys. 2019, 21, 5999–6010. 10.1039/C9CP00587K. 30809621

[ref11] Docampo-Álvarez B.; Gómez-González V.; Méndez-Morales T.; Rodríguez J. R.; Cabeza O.; Turmine M.; Gallego L. J.; Varela L. M. The Effect of Alkyl Chain Length on the Structure and Thermodynamics of Protic-Aprotic Ionic Liquid Mixtures: A Molecular Dynamics Study. Phys. Chem. Chem. Phys. 2018, 20, 9938–9949. 10.1039/C8CP00575C. 29619465

[ref12] Herrera C.; Atilhan M.; Aparicio S. A Theoretical Study on Mixtures of Amino Acid-Based Ionic Liquids. Phys. Chem. Chem. Phys. 2018, 20, 10213–10223. 10.1039/C7CP08533H. 29594303

[ref13] Brooks N. J.; Castiglione F.; Doherty C. M.; Dolan A.; Hill A. J.; Hunt P. A.; Matthews R. P.; Mauri M.; Mele A.; Simonutti R.; et al. Linking the Structures, Free Volumes, and Properties of Ionic Liquid Mixtures. Chem. Sci. 2017, 8, 6359–6374. 10.1039/C7SC01407D. 29619199 PMC5859882

[ref14] Lepre L. F.; Szala-Bilnik J.; Padua A. A. H.; Traïkia M.; Ando R. A.; Costa Gomes M. F. Tailoring the Properties of Acetate-Based Ionic Liquids Using the Tricyanomethanide Anion. Phys. Chem. Chem. Phys. 2016, 18, 23285–23295. 10.1039/C6CP04651G. 27498753

[ref15] Hollóczki O.; Macchiagodena M.; Weber H.; Thomas M.; Brehm M.; Stark A.; Russina O.; Triolo A.; Kirchner B. Triphilic Ionic-Liquid Mixtures: Fluorinated and Non-Fluorinated Aprotic Ionic-Liquid Mixtures. ChemPhysChem 2015, 16, 3325–3333. 10.1002/cphc.201500473. 26305804 PMC4641458

[ref16] Bayley P. M.; Best A. S.; MacFarlane D. R.; Forsyth M. Transport Properties and Phase Behaviour in Binary and Ternary Ionic Liquid Electrolyte Systems of Interest in Lithium Batteries. ChemPhysChem 2011, 12, 823–827. 10.1002/cphc.201000909. 21264997

[ref17] Navia P.; Troncoso J.; Romaní L. Viscosities for Ionic Liquid Binary Mixtures with a Common Ion. J. Solution Chem. 2008, 37, 677–688. 10.1007/s10953-008-9260-8.

[ref18] Song D.; Chen J. Density and Viscosity Data for Mixtures of Ionic Liquids with a Common Anion. J. Chem. Eng. Data 2014, 59, 257–262. 10.1021/je400332j.

[ref19] Almeida H. F. D.; Canongia Lopes J. N.; Rebelo L. P. N.; Coutinho J. A. P.; Freire M. G.; Marrucho I. M. Densities and Viscosities of Mixtures of Two Ionic Liquids Containing a Common Cation. J. Chem. Eng. Data 2016, 61, 2828–2843. 10.1021/acs.jced.6b00178.

[ref20] Cosby T.; Kapoor U.; Shah J. K.; Sangoro J. Mesoscale Organization and Dynamics in Binary Ionic Liquid Mixtures. J. Phys. Chem. Lett. 2019, 6274–6280. 10.1021/acs.jpclett.9b02478. 31560210

[ref21] Marullo S.; D’Anna F.; Campodonico P. R.; Noto R. Ionic Liquid Binary Mixtures: How Different Factors Contribute to Determine Their Effect on the Reactivity. RSC Adv. 2016, 6, 90165–90171. 10.1039/C6RA12836J.

[ref22] Cha S.; Kim D. Change of Hydrogen Bonding Structure in Ionic Liquid Mixtures by Anion Type. J. Chem. Phys. 2018, 148, 193827 10.1063/1.5010067. 30307177

[ref23] Payal R. S.; Balasubramanian S. Homogenous Mixing of Ionic Liquids: Molecular Dynamics Simulations. Phys. Chem. Chem. Phys. 2013, 15, 21077–21083. 10.1039/c3cp53492h. 24220142

[ref24] Matthews R. P.; Villar-Garcia I. J.; Weber C. C.; Griffith J.; Cameron F.; Hallett J. P.; Hunt P. A.; Welton T. A Structural Investigation of Ionic Liquid Mixtures. Phys. Chem. Chem. Phys. 2016, 18, 8608–8624. 10.1039/C6CP00156D. 26947103

[ref25] Nemoto F.; Kofu M.; Nagao M.; Ohishi K.; Takata S.-I.; Suzuki J.-I.; Yamada T.; Shibata K.; Ueki T.; Kitazawa Y.; et al. Neutron Scattering Studies on Short- and Long-Range Layer Structures and Related Dynamics in Imidazolium-Based Ionic Liquids. J. Chem. Phys. 2018, 149, 054502 10.1063/1.5037217. 30089384

[ref26] Andanson J.-M.; Beier M. J.; Baiker A. Binary Ionic Liquids with a Common Cation: Insight into Nanoscopic Mixing by Infrared Spectroscopy. J. Phys. Chem. Lett. 2011, 2, 2959–2964. 10.1021/jz201323a.

[ref27] Kunze M.; Jeong S.; Paillard E.; Winter M.; Passerini S. Melting Behavior of Pyrrolidinium-Based Ionic Liquids and Their Binary Mixtures. J. Phys. Chem. C 2010, 114, 12364–12369. 10.1021/jp103746k. 20058901

[ref28] Shimizu K.; Tariq M.; Rebelo L. P. N.; Canongia Lopes J. N. Binary Mixtures of Ionic Liquids with a Common Ion Revisited: A Molecular Dynamics Simulation Study. J. Mol. Liq. 2010, 153, 52–56. 10.1016/j.molliq.2009.07.012.

[ref29] Annat G.; Forsyth M.; MacFarlane D. R. Ionic Liquid Mixtures-Variations in Physical Properties and Their Origins in Molecular Structure. J. Phys. Chem. B 2012, 116, 8251–8258. 10.1021/jp3012602. 22759206

[ref30] Bruce D. W.; Cabry C. P.; Canongia Lopes J. N.; Costen M. L.; D’Andrea L.; Grillo I.; Marshall B. C.; McKendrick K. G.; Minton T. K.; Purcell S. M.; et al. Nanosegregation and Structuring in the Bulk and at the Surface of Ionic-Liquid Mixtures. J. Phys. Chem. B 2017, 121, 6002–6020. 10.1021/acs.jpcb.7b01654. 28459567

[ref31] Kanakubo M.; Makino T.; Umecky T. CO2 Solubility in and Physical Properties for Ionic Liquid Mixtures of 1-Butyl-3-Methylimidazolium Acetate and 1-Butyl-3-Methylimidazolium Bis(Trifluoromethanesulfonyl)Amide. J. Mol. Liq. 2016, 217, 112–119. 10.1016/j.molliq.2016.02.018.

[ref32] Xiao D.; Rajian J. R.; Li S.; Bartsch R. A.; Quitevis E. L. Additivity in the Optical Kerr Effect Spectra of Binary Ionic Liquid Mixtures: Implications for Nanostructural Organization. J. Phys. Chem. B 2006, 110, 16174–16178. 10.1021/jp063740o. 16913736

[ref33] Russina O.; Lo Celso F.; Plechkova N. V.; Triolo A. Emerging Evidences of Mesoscopic-Scale Complexity in Neat Ionic Liquids and Their Mixtures. J. Phys. Chem. Lett. 2017, 8, 1197–1204. 10.1021/acs.jpclett.6b02811. 28234000

[ref34] Russina O.; Triolo A. New Experimental Evidence Supporting the Mesoscopic Segregation Model in Room Temperature Ionic Liquids. Faraday Discuss. 2012, 154, 97–109. 10.1039/C1FD00073J. 22455016

[ref35] Bini R.; Bortolini O.; Chiappe C.; Pieraccini D.; Siciliano T. Development of Cation/Anion “Interaction” Scales for Ionic Liquids through ESI-MS Measurements. J. Phys. Chem. B 2007, 111, 598–604. 10.1021/jp0663199. 17228918

[ref36] Nanda R.; Damodaran K. A Review of NMR Methods Used in the Study of the Structure and Dynamics of Ionic Liquids. Magn. Reson. Chem. 2018, 56, 62–72. 10.1002/mrc.4666. 28921712

[ref37] Giernoth R. NMR Spectroscopy in Ionic Liquds. Top. Curr. Chem. 2008, 290, 263–283. 10.1007/128_2008_37. 21107800

[ref38] D’Agostino C.; Mantle M. D.; Mullan C. L.; Hardacre C.; Gladden L. F. Diffusion, Ion Pairing and Aggregation in 1-Ethyl-3-Methylimidazolium-Based Ionic Liquids Studied by ^1^H and ^19^F PFG NMR: Effect of Temperature, Anion and Glucose Dissolution. ChemPhysChem 2018, 19, 1081–1088. 10.1002/cphc.201701354. 29385314

[ref39] Weingärtner H. Understanding Ionic Liquids at the Molecular Level: Facts, Problems, and Controversies. Angew. Chem., Int. Ed. 2008, 47, 654–670. 10.1002/anie.200604951. 17994652

[ref40] Holbrey J. D.; Seddon K. R. The phase behaviour of 1-alkyl-3-methylimidazolium tetrafluoroborates; ionic liquids and ionic liquid crystals. J. Chem. Soc., Dalton Trans. 1999, 2133–2140. 10.1039/a902818h.

[ref41] Carper W. R.; Wahlbeck P. G.; Dölle A. 13C NMR Relaxation Rates: Separation of Dipolar and Chemical Shift Anisotropy Effects. J. Phys. Chem. A 2004, 108, 6096–6099. 10.1021/jp031300g.

[ref42] Antony J. H.; Mertens D.; Dölle A.; Wasserscheid P.; Carper W. R. Molecular Reorientational Dynamics of the Neat Ionic Liquid 1-Butyl-3-Methylimidazolium Hexafluorophosphate by Measurement of ^13^C Nuclear Magnetic Relaxation Data. ChemPhysChem 2003, 4, 588–594. 10.1002/cphc.200200603. 12836480

[ref43] Imanari M.; Fujii K.; Mukai T.; Mizushima N.; Seki H.; Nishikawa K. Anion and Cation Dynamics of Sulfonylamide-Based Ionic Liquids and the Solid-Liquid Transitions. Phys. Chem. Chem. Phys. 2015, 17, 8750–8757. 10.1039/C5CP00302D. 25738430

[ref44] Shimizu Y.; Wachi Y.; Fujii K.; Imanari M.; Nishikawa K. NMR Study on Ion Dynamics and Phase Behavior of a Piperidinium-Based Room-Temperature Ionic Liquid: 1-Butyl-1-Methylpiperidinium Bis(Fluorosulfonyl)Amide. J. Phys. Chem. B 2016, 120, 5710–5719. 10.1021/acs.jpcb.6b04095. 27281062

[ref45] Allen J. J.; Bowser S. R.; Damodaran K. Molecular Interactions in the Ionic Liquid Emim Acetate and Water Binary Mixtures Probed via NMR Spin Relaxation and Exchange Spectroscopy. Phys. Chem. Chem. Phys. 2014, 16, 8078–8085. 10.1039/C3CP55384A. 24654003

[ref46] Alam T. M.; Dreyer D. R.; Bielwaski C. W.; Ruoff R. S. Measuring Molecular Dynamics and Activation Energies for Quaternary Acyclic Ammonium and Cyclic Pyrrolidinium Ionic Liquids Using 14N NMR Spectroscopy. J. Phys. Chem. A 2011, 115, 4307–4316. 10.1021/jp200630k. 21456554

[ref47] Bystrov S. S.; Matveev V. V.; Chernyshev Y. S.; Balevičius V.; Chizhik V. I. Molecular Mobility in a Set of Imidazolium-Based Ionic Liquids [Bmim]^+^A^–^ by the NMR-Relaxation Method. J. Phys. Chem. B 2019, 123, 2362–2372. 10.1021/acs.jpcb.8b11250. 30779569

[ref48] Rumble C. A.; Kaintz A.; Yadav S. K.; Conway B.; Araque J. C.; Baker G. A.; Margulis C.; Maroncelli M. Rotational Dynamics in Ionic Liquids from NMR Relaxation Experiments and Simulations: Benzene and 1-Ethyl-3-Methylimidazolium. J. Phys. Chem. B 2016, 120, 9450–9467. 10.1021/acs.jpcb.6b06715. 27509215

[ref49] Allen J. J.; Schneider Y.; Kail B. W.; Luebke D. R.; Nulwala H.; Damodaran K. Nuclear Spin Relaxation and Molecular Interactions of a Novel Triazolium-Based Ionic Liquid. J. Phys. Chem. B 2013, 117, 3877–3883. 10.1021/jp401188g. 23470049

[ref50] Endo T.; Murata H.; Imanari M.; Mizushima N.; Seki H.; Sen S.; Nishikawa K. A Comparative Study of the Rotational Dynamics of PF_6_- Anions in the Crystals and Liquid States of 1-Butyl-3- Methylimidazolium Hexafluorophosphate: Results from ^31^P NMR Spectroscopy. J. Phys. Chem. B 2013, 117, 326–332. 10.1021/jp310947c. 23241082

[ref51] Remsing R. C.; Hernandez G.; Swatloski R. P.; Massefski W. W.; Rogers R. D.; Moyna G. Solvation of Carbohydrates in N,N’-Dialkylimidazolium Ionic Liquids: A Multinuclear NMR Spectroscopy Study. J. Phys. Chem. B 2008, 112, 11071–11078. 10.1021/jp8042895. 18693699

[ref52] Strate A.; Neumann J.; Overbeck V.; Bonsa A.-M.; Michalik D.; Paschek D.; Ludwig R. Rotational and Translational Dynamics and Their Relation to Hydrogen Bond Lifetimes in an Ionic Liquid by Means of NMR Relaxation Time Experiments and Molecular Dynamics Simulation. J. Chem. Phys. 2018, 148, 193843 10.1063/1.5011804. 30307203

[ref53] Nakamura K.; Shikata T. Systematic Dielectric and NMR Study of the Ionic Liquid 1-Alkyl-3-Methyl Imidazolium. ChemPhysChem 2010, 11, 285–294. 10.1002/cphc.200900642. 19998306

[ref54] Hayamizu K.; Tsuzuki S.; Seki S.; Umebayashi Y. Nuclear Magnetic Resonance Studies on the Rotational and Translational Motions of Ionic Liquids Composed of 1-Ethyl-3-Methylimidazolium Cation and Bis(Trifluoromethanesulfonyl)Amide and Bis(Fluorosulfonyl)Amide Anions and Their Binary Systems Including Lithium salts. J. Chem. Phys. 2011, 135, 084505 10.1063/1.3625923. 21895197

[ref55] Klimavicius V.; Bacevicius V.; Gdaniec Z.; Balevicius V. Pulsed- Field Gradient 1H NMR Study of Diffusion and Self-Aggregation of Long-Chain Imidazolium-Based Ionic Liquids. J. Mol. Liq. 2015, 210, 223–226. 10.1016/j.molliq.2015.05.034.

[ref56] Gordon P. G.; Brouwer D. H.; Ripmeester J. A. Probing the Local Structure of Pure Ionic Liquid Salts with Solid- and Liquid-State NMR. ChemPhysChem 2010, 11, 260–268. 10.1002/cphc.200900624. 19924756

[ref57] Klimavicius V.; Gdaniec Z.; Balevicius V. Very Short NMR Relaxation Times of Anions in Ionic Liquids: New Pulse Sequence to Eliminate the Acoustic Ringing. Spectrochim. Acta, Part A 2014, 132, 879–883. 10.1016/j.saa.2014.04.140. 24938418

[ref58] Becher M.; Steinrücken E.; Vogel M. On the Relation between Reorientation and Diffusion in Glass-Forming Ionic Liquids with Micro-Heterogeneous Structures. J. Chem. Phys. 2019, 151, 194503 10.1063/1.5128420. 31757165

[ref59] Endo T.; Murata H.; Imanari M.; Mizushima N.; Seki H.; Nishikawa K. NMR Study of Cation Dynamics in Three Crystalline States of 1-Butyl-3-Methylimidazolium Hexafluorophosphate Exhibiting Crystal Polymorphism. J. Phys. Chem. B 2012, 116, 3780–3788. 10.1021/jp300636s. 22380424

[ref60] Hayamizu K.; Tsuzuki S.; Seki S.; Fujii K.; Suenaga M.; Umebayashi Y. Studies on the Translational and Rotational Motions of Ionic Liquids Composed of N -Methyl- N -Propyl-Pyrrolidinium (P13) Cation and Bis(Trifluoromethanesulfonyl)Amide and Bis(Fluorosulfonyl)Amide Anions and Their Binary Systems Including Lithium Salts. J. Chem. Phys. 2010, 133, 194505 10.1063/1.3505307. 21090866

[ref61] Hayamizu K.; Tsuzuki S.; Seki S.; Umebayashi Y. Multinuclear NMR Studies on Translational and Rotational Motion for Two Ionic Liquids Composed of BF_4_ Anion. J. Phys. Chem. B 2012, 116, 11284–11291. 10.1021/jp306146s. 22908967

[ref62] Annat G.; MacFarlane D. R.; Forsyth M. Transport Properties in Ionic Liquids and Ionic Liquid Mixtures: The Challenges of NMR Pulsed Field Gradient Diffusion Measurements. J. Phys. Chem. B 2007, 111, 9018–9024. 10.1021/jp072737h. 17608524

[ref63] Castiglione F.; Moreno M.; Raos G.; Famulari A.; Mele A.; Appetecchi G. B.; Passerini S. Structural Organization and Transport Properties of Novel Pyrrolidinium-Based Ionic Liquids with Perfluoroalkyl Sulfonylimide Anions. J. Phys. Chem. B 2009, 113, 10750–10759. 10.1021/jp811434e. 19601598

[ref64] Judeinstein P.; Iojoiu C.; Sanchez J.-Y.; Ancian B. Proton Conducting Ionic Liquid Organization as Probed by NMR: Self-Diffusion Coefficients and Heteronuclear Correlations. J. Phys. Chem. B 2008, 112, 3680–3683. 10.1021/jp711298g. 18321091

[ref65] Alam T. M.; Dreyer D. R.; Bielawski C. W.; Ruoff R. S. Combined Measurement of Translational and Rotational Diffusion in Quaternary Acyclic Ammonium and Cyclic Pyrrolidinium Ionic Liquids. J. Phys. Chem. B 2013, 117, 1967–1977. 10.1021/jp3111953. 23327476

[ref66] Martinelli A.; Maréchal M.; Östlund Å.; Cambedouzou J. Insights into the Interplay between Molecular Structure and Diffusional Motion in 1-Alkyl-3-Methylimidazolium Ionic Liquids: A Combined PFG NMR and X-Ray Scattering Study. Phys. Chem. Chem. Phys. 2013, 15, 5510–5517. 10.1039/c3cp00097d. 23455003

[ref67] Nanda R. Thermal Dynamics of Lithium Salt Mixtures of Ionic Liquid in Water by PGSE NMR Spectroscopy. RSC Adv. 2016, 6, 36394–36406. 10.1039/C6RA00891G.

[ref68] Noda A.; Hayamizu K.; Watanabe M. Pulsed-Gradient Spin-Echo ^1^H and ^19^F NMR Ionic Diffusion Coefficient, Viscosity, and Ionic Conductivity of Non-Chloroaluminate Room-Temperature Ionic Liquids. J. Phys. Chem. B 2001, 105, 4603–4610. 10.1021/jp004132q.

[ref69] Sangoro J.; Iacob C.; Serghei A.; Naumov S.; Galvosas P.; Kärger J.; Wespe C.; Bordusa F.; Stoppa A.; Hunger J.; et al. Electrical Conductivity and Translational Diffusion in the 1-Butyl-3-Methylimidazolium Tetrafluoroborate Ionic Liquid. J. Chem. Phys. 2008, 128, 214509 10.1063/1.2921796. 18537435

[ref70] Chiappe C.; Sanzone A.; Mendola D.; Castiglione F.; Famulari A.; Raos G.; Mele A. Pyrazolium- versus Imidazolium-Based Ionic Liquids: Structure, Dynamics and Physicochemical Properties. J. Phys. Chem. B 2013, 117, 668–676. 10.1021/jp3107793. 23252760

[ref71] Casalegno M.; Raos G.; Appetecchi G. B.; Passerini S.; Castiglione F.; Mele A. From Nanoscale to Microscale: Crossover in the Diffusion Dynamics within Two Pyrrolidinium-Based Ionic Liquids. J. Phys. Chem. Lett. 2017, 8, 5196–5202. 10.1021/acs.jpclett.7b02431. 28976762

[ref72] Mantz R. A.; Trulove P. C.; Carlin R. T.; Osteryoung R. A. ROESY NMR of Basic Ambient-Temperature Chloroaluminate Ionic Liquids. Inorg. Chem. 1995, 34, 3846–3847. 10.1021/ic00118a042. 11669690

[ref73] Mele A.; Romanò G.; Giannone M.; Ragg E.; Fronza G.; Raos G.; Marcon V. The Local Structure of Ionic Liquids: Cation-Cation NOE Interactions and Internuclear Distances in Neat [BMIM][BF_4_] and [BDMIM][BF_4_]. Angew. Chem., Int. Ed. 2006, 45, 1123–1126. 10.1002/anie.200503745. 16411265

[ref74] Lingscheid Y.; Arenz S.; Giernoth R. Heteronuclear NOE Spectroscopy of Ionic Liquids. ChemPhysChem 2012, 13, 261–266. 10.1002/cphc.201100622. 22002917

[ref75] Dupont J.; Suarez P. A. Z.; De Souza R. F.; Burrow R. A.; Kintzinger J.-P. C-H-π Interactions in 1-n-Butyl-3-Methylimidazolium Tetraphenylborate Molten Salt: Solid and Solution Structures. Chem. Eur. J. 2000, 6, 2377–2381. 10.1002/1521-3765(20000703)6:13<2377::AID-CHEM2377>3.0.CO;2-L. 10939740

[ref76] Gutel T.; Santini C. C.; Pádua A. A. H.; Fenet B.; Chauvin Y.; Canongia Lopes J. N.; Bayard F.; Costa Gomes M. F.; Pensado A. S. Interaction between the π-System of Toluene and the Imidazolium Ring of Ionic Liquids: A Combined NMR and Molecular Simulation Study. J. Phys. Chem. B 2009, 113, 170–177. 10.1021/jp805573t. 19195088

[ref77] Gabl S.; Steinhauser O.; Weingärtner H. From Short-Range to Long-Range Intermolecular NOEs in Ionic Liquids: Frequency Does Matter. Angew. Chem., Int. Ed. 2013, 52, 9242–9246. 10.1002/anie.201302712. 23776138

[ref78] Castiglione F.; Appetecchi G. B.; Passerini S.; Panzeri W.; Indelicato S.; Mele A. Multiple Points of View of Heteronuclear NOE: Long Range vs Short Range Contacts in Pyrrolidinium Based Ionic Liquids in the Presence of Li Salts. J. Mol. Liq. 2015, 210, 215–222. 10.1016/j.molliq.2015.05.036.

[ref79] Martin P.-A.; Chen F.; Forsyth M.; Deschamps M.; O’Dell L. A. Correlating Intermolecular Cross-Relaxation Rates with Distances and Coordination Numbers in Ionic Liquids. J. Phys. Chem. Lett. 2018, 9, 7072–7078. 10.1021/acs.jpclett.8b03021. 30395468

[ref80] Bax A.; Grzesiek S. ROESY. Encycl. Magn. Reson. 2007, 1–10. 10.1002/9780470034590.emrstm0473.

[ref81] Martin P.-A.; Salager E.; Forsyth M.; O’Dell L. A.; Deschamps M. On the Measurement of Intermolecular Heteronuclear Cross Relaxation Rates in Ionic Liquids. Phys. Chem. Chem. Phys. 2018, 20, 13357–13364. 10.1039/C8CP00911B. 29718051

[ref82] Cesare Marincola F.; Piras C.; Russina O.; Gontrani L.; Saba G.; Lai A. NMR Investigation of Imidazolium-Based Ionic Liquids and Their Aqueous Mixtures. ChemPhysChem 2012, 13, 1339–1346. 10.1002/cphc.201100810. 22266801

[ref83] Khatun S.; Castner E. W. Jr. Ionic Liquid-Solute Interactions Studied by 2D NOE NMR Spectroscopy. J. Phys. Chem. B 2015, 119, 9225–9235. 10.1021/jp509861g. 25402509

[ref84] Lee H. Y.; Shirota H.; Castner E. W. Jr. Differences in Ion Interactions for Isoelectronic Ionic Liquid Homologs. J. Phys. Chem. Lett. 2013, 4, 1477–1483. 10.1021/jz400465x. 26282302

