# Supplementary material for: Anions as Dynamic Probes for Ionic Liquid Mixtures
Source: J Phys Chem B. 2020 Mar 18;124(14):2879–91. doi: 10.1021/acs.jpcb.0c00026 (PMC7997561; doi:10.1021/acs.jpcb.0c00026)
Supplement: Supplementary file 1 — jp0c00026_si_001.pdf [file jp0c00026_si_001.pdf]

Supplementary Material

Anions as Dynamic Probes for Ionic Liquid Mixtures

Maria Enrica Di Pietro,[*,a] Franca Castiglione,*[a] and Andrea Mele.*[*,a,b]

[a] Department of Chemistry, Materials and Chemical Engineering "G. Natta", Politecnico di Milano, Piazza L. da Vinci 32, 20133 Milano, Italy.

[b] Istituto di Scienze e Tecnologie Chimiche (SCITEC-CNR), Via A. Corti 12, 20133 Milano, Italy.

Table of contents

Brief survey of reported viscosity data S3

1D ^{1}H NMR spectra at 325 K of BF_4- and TFSI-mixtures S5

T_1 and T_2 relaxation times and T_1/T_2 ratio measured at given sites in all samples S6

Arrhenius plots and apparent activation energies obtained from temperature-dependent T_1 data S14

Self-diffusion coefficients measured in all samples S17

Arrhenius plots and apparent activation energies obtained from temperature-dependent D data S20

ROESY spectra S22

Integrated peak volume of HOESY cross peaks S24

Theoretical background of relaxation and diffusion analysis S25

References S27

Brief survey of reported viscosity data

Bruce *et al.* recently reported a detailed investigation of [C_{12}mim][C_2mim][TFSI] mixtures.[1] According to their data, deviation from ideal mixing behaviour are seen when using both the Grinberg-Nissan and the Katti-Chaudhri mixing laws, which are widely accepted to describe the mixing behaviour of IL mixtures. As reported in Fig. S1a, maximum deviations were of 17% at the lowest temperature (293 K).

Viscosity data for [C_6mim][C_2mim][BF_4] mixtures have been reported by Navia *et al*. and Song *et al.*.[2,3] In both cases, authors concluded that the Grinberg-Nissan and the Katti-Chaudhri mixing laws give good results, confirming that IL mixtures do not deviate by a large amount from ideal behaviour. Experimental data from the literature are plotted in Fig. S1, together with the percentage of deviation as calculated from Bruce *et al.*. It can be observed that deviations up to 11% exist, which were judged quite small by the authors.

Regardless of the threshold after which a deviation should be considered negligible or significant, the main point here is that, even if a certain amount of deviation from the ideal mixing behaviour is observed in terms of viscosity, a general trend is seen: viscosity increases with increasing of the bigger cation. This is after all what one would expect from common sense and has been observed qualitatively during sample preparation and in terms of linewidth in NMR spectra.

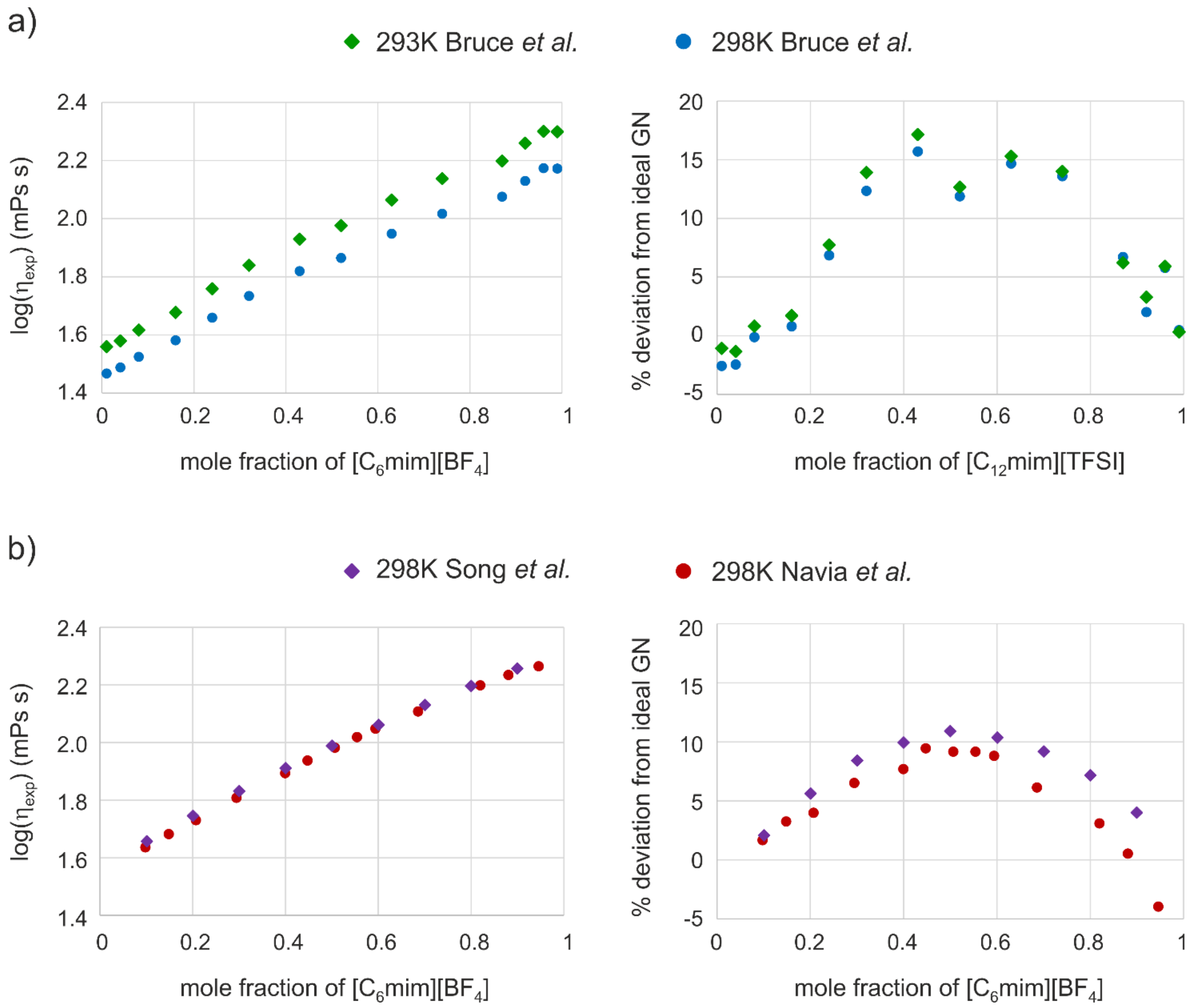

Figure S1. Experimental viscosities η_{exp} and percentage deviation from the Grunberg–Nissan (GN) mixing law as a function of composition for (a) $[C_{12}mim]_x[C_2mim]_{1-x}[TFSI]$ mixtures (from data of ref [1]), and (b) $[C_6mim]_x[C_2mim]_{1-x}[BF_4]$ mixtures (from data of refs [2] and [3]).

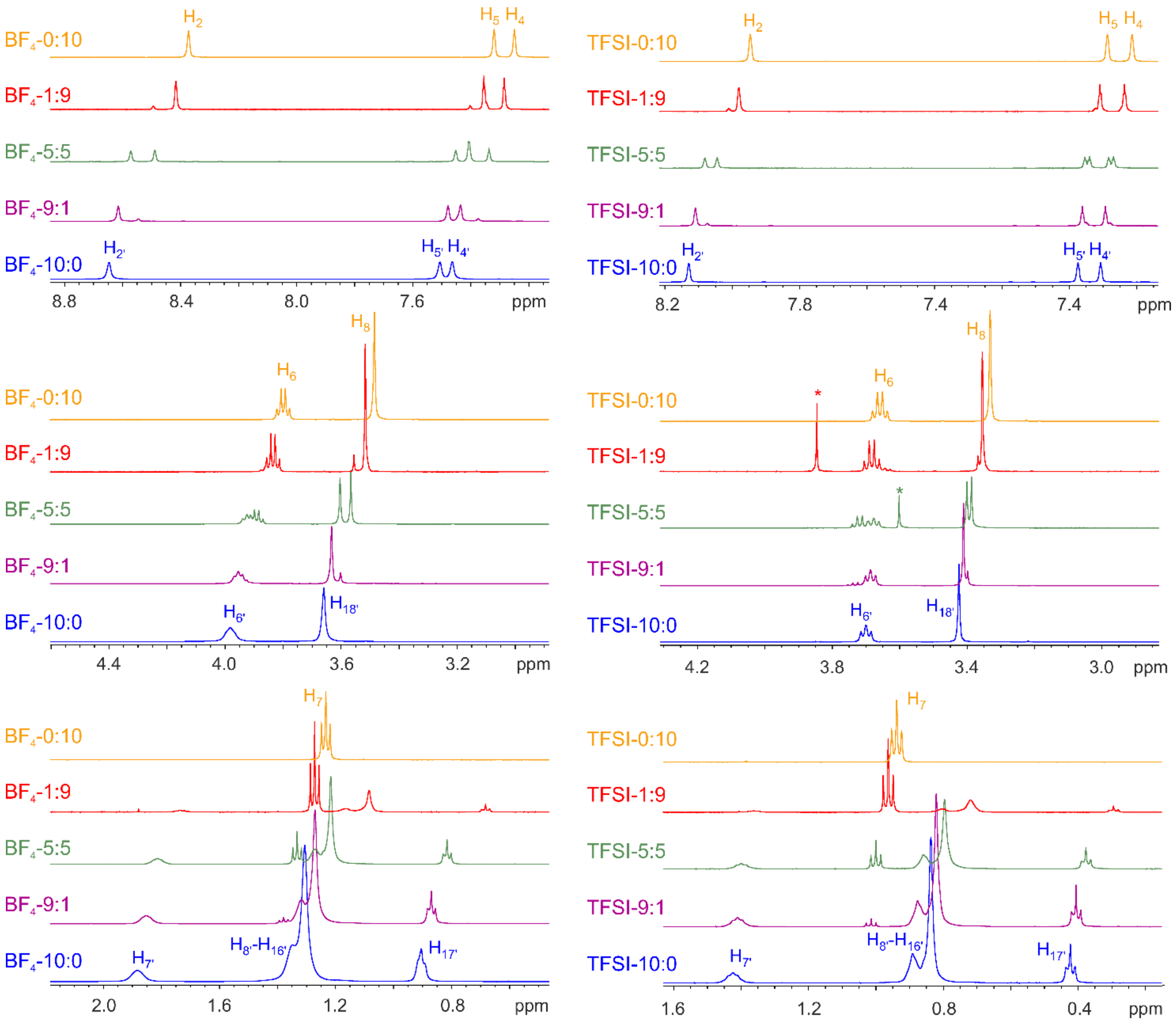

Figure S2. 1D ^{1}H NMR spectra at 325 K of BF_4-mixtures (on the left) and TFSI-mixtures (on the right). Peaks labelled with an asterisk correspond to an impurity in the capillary.

Table S1. T_1 and T_2 relaxation times and T_1/T_2 ratio measured for proton $H_{2'}$ of the cation $[C_{12}mim]^+$ in all samples. T_1 and T_2 are estimated to be accurate within ±1% and ±5%, respectively.

	**T_1 (s)**							
**T (K)**	**BF_4-10:0**	**BF_4-9:1**	**BF_4-5:5**	**BF_4-1:9**	**TFSI-10:0**	**TFSI-9:1**	**TFSI-5:5**	**TFSI-1:9**
**325**	0.97	0.98	1.07	1.12	1.36	1.43	1.524	1.80
**320**	0.95	0.97	1.06	1.11	1.27	1.36	1.447	1.74
**315**		0.92	1.02	1.07	1.21	1.29	1.387	1.58
**305**		0.84	0.93	0.98	1.08	1.16	1.265	1.48
**300**		0.80	0.90	0.97	1.02	1.09	1.20	1.38
	**T_2 (s)**							
**T (K)**	**BF_4-10:0**	**BF_4-9:1**	**BF_4-5:5**	**BF_4-1:9**	**TFSI-10:0**	**TFSI-9:1**	**TFSI-5:5**	**TFSI-1:9**
**325**	0.17	0.21	0.35	0.55	0.62	0.68	0.80	0.96
**320**	0.14	0.16	0.28	0.42	0.55	0.56	0.67	0.93
**315**		0.12	0.20	0.32	0.46	0.49	0.64	0.73
**305**		0.07	0.15	0.28	0.31	0.35	0.47	0.71
**300**		0.05	0.13	0.19	0.23	0.28	0.41	0.60
	**T_1 / T_2**							
**T (K)**	**BF_4-10:0**	**BF_4-9:1**	**BF_4-5:5**	**BF_4-1:9**	**TFSI-10:0**	**TFSI-9:1**	**TFSI-5:5**	**TFSI-1:9**
**325**	5.8	4.7	3.1	2.0	2.2	2.1	1.9	1.9
**320**	7.0	6.2	3.8	2.6	2.3	2.4	2.2	1.9
**315**		8.0	5.1	3.3	2.6	2.6	2.2	2.2
**305**		11.6	6.1	3.4	3.5	3.4	2.7	2.1
**300**		15.1	7.2	5.0	4.4	3.9	3.0	2.3

Table S2. T_1 and T_2 relaxation times and T_1/T_2 ratio measured for proton H_2 of the cation $[C_2mim]^+$ in all samples. T_1 and T_2 are estimated to be accurate within ±1% and ±5%, respectively.

	**T_1 (s)**							
**T (K)**	**BF_4-9:1**	**BF_4-5:5**	**BF_4-1:9**	**BF_4-0:10**	**TFSI-9:1**	**TFSI-5:5**	**TFSI-1:9**	**TFSI-0:10**
**325**	1.21	1.32	1.46	1.54	1.73	1.99	2.42	2.52
**320**	1.13	1.31	1.42	1.47	1.66	1.86	2.25	2.32
**315**	1.07	1.25	1.32	1.39	1.56	1.76	2.10	2.17
**305**	0.99	1.18	1.17	1.24	1.36	1.56	1.85	1.81
**300**	0.96	1.12	1.17	1.16	1.30	1.49	1.76	1.78
	**T_2 (s)**							
**T (K)**	**BF_4-9:1**	**BF_4-5:5**	**BF_4-1:9**	**BF_4-0:10**	**TFSI-9:1**	**TFSI-5:5**	**TFSI-1:9**	**TFSI-0:10**
**325**	0.34	0.51	0.60	0.70	0.95	1.16	1.43	1.56
**320**	0.30	0.45	0.51	0.67	0.82	1.04	1.34	1.43
**315**	0.21	0.41	0.48	0.55	0.71	0.97	1.25	1.31
**305**	0.15	0.29	0.41	0.51	0.51	0.78	1.08	1.19
**300**	0.13	0.24	0.32	0.41	0.44	0.69	0.97	1.05
	**T_1 / T_2**							
**T (K)**	**BF_4-9:1**	**BF_4-5:5**	**BF_4-1:9**	**BF_4-0:10**	**TFSI-9:1**	**TFSI-5:5**	**TFSI-1:9**	**TFSI-0:10**
**325**	3.6	2.6	2.4	2.2	1.8	1.7	1.7	1.6
**320**	3.8	2.9	2.8	2.2	2.0	1.8	1.7	1.6
**315**	5.2	3.1	2.8	2.5	2.2	1.8	1.7	1.7
**305**	6.5	4.0	2.9	2.4	2.7	2.0	1.7	1.5
**300**	7.4	4.6	3.7	2.8	3.0	2.2	1.8	1.7

Table S3. T_1 and T_2 relaxation times and T_1/T_2 ratio measured for fluorine of the anion $[BF_4]^-$ or $[TFSI]^-$ in all samples. T_1 and T_2 are estimated to be accurate within ±1% and ±5%, respectively.

	**T_1 (s)**									
**T (K)**	**BF_4- 10:0**	**BF_4- 9:1**	**BF_4- 5:5**	**BF_4- 1:9**	**BF_4- 0:10**	**TFSI- 10:0**	**TFSI- 9:1**	**TFSI- 5:5**	**TFSI- 1:9**	**TFSI- 0:10**
**325**	1.36	1.45	1.81	2.13	2.21	0.82	0.87	0.97	1.16	1.22
**320**	1.32	1.41	1.79	2.10	2.13	0.77	0.81	0.89	1.07	1.12
**315**	1.17	1.31	1.67	1.99	2.05	0.71	0.75	0.82	0.97	1.02
**305**	1.13	1.17	1.43	1.75	1.85	0.62	0.65	0.70	0.82	0.85
**300**	1.09	1.12	1.41	1.69	1.74	0.57	0.61	0.65	0.76	0.78
	**T_2 (s)**									
**T (K)**	**BF_4- 10:0**	**BF_4- 9:1**	**BF_4- 5:5**	**BF_4- 1:9**	**BF_4- 0:10**	**TFSI- 10:0**	**TFSI- 9:1**	**TFSI- 5:5**	**TFSI- 1:9**	**TFSI- 0:10**
**325**	0.40	0.52	0.85	1.16	1.19	0.38	0.42	0.59	0.83	0.88
**320**	0.32	0.43	0.79	1.07	1.17	0.32	0.36	0.52	0.74	0.83
**315**	0.22	0.34	0.67	1.04	1.07	0.27	0.31	0.44	0.67	0.73
**305**	0.13	0.20	0.43	0.81	0.92	0.18	0.20	0.33	0.51	0.58
**300**	0.10	0.15	0.38	0.77	0.83	0.14	0.16	0.27	0.45	0.51
	**T_1 / T_2**									
**T (K)**	**BF_4- 10:0**	**BF_4- 9:1**	**BF_4- 5:5**	**BF_4- 1:9**	**BF_4- 0:10**	**TFSI- 10:0**	**TFSI- 9:1**	**TFSI- 5:5**	**TFSI- 1:9**	**TFSI- 0:10**
**325**	3.4	2.8	2.1	1.8	1.9	2.2	2.1	1.6	1.4	1.4
**320**	4.2	3.3	2.2	2.0	1.8	2.4	2.2	1.7	1.4	1.4
**315**	5.3	3.9	2.5	1.9	1.9	2.6	2.5	1.9	1.5	1.4
**305**	8.4	5.7	3.3	2.2	2.0	3.5	3.2	2.2	1.6	1.5
**300**	11.1	7.4	3.8	2.2	2.1	4.1	3.7	2.4	1.7	1.5

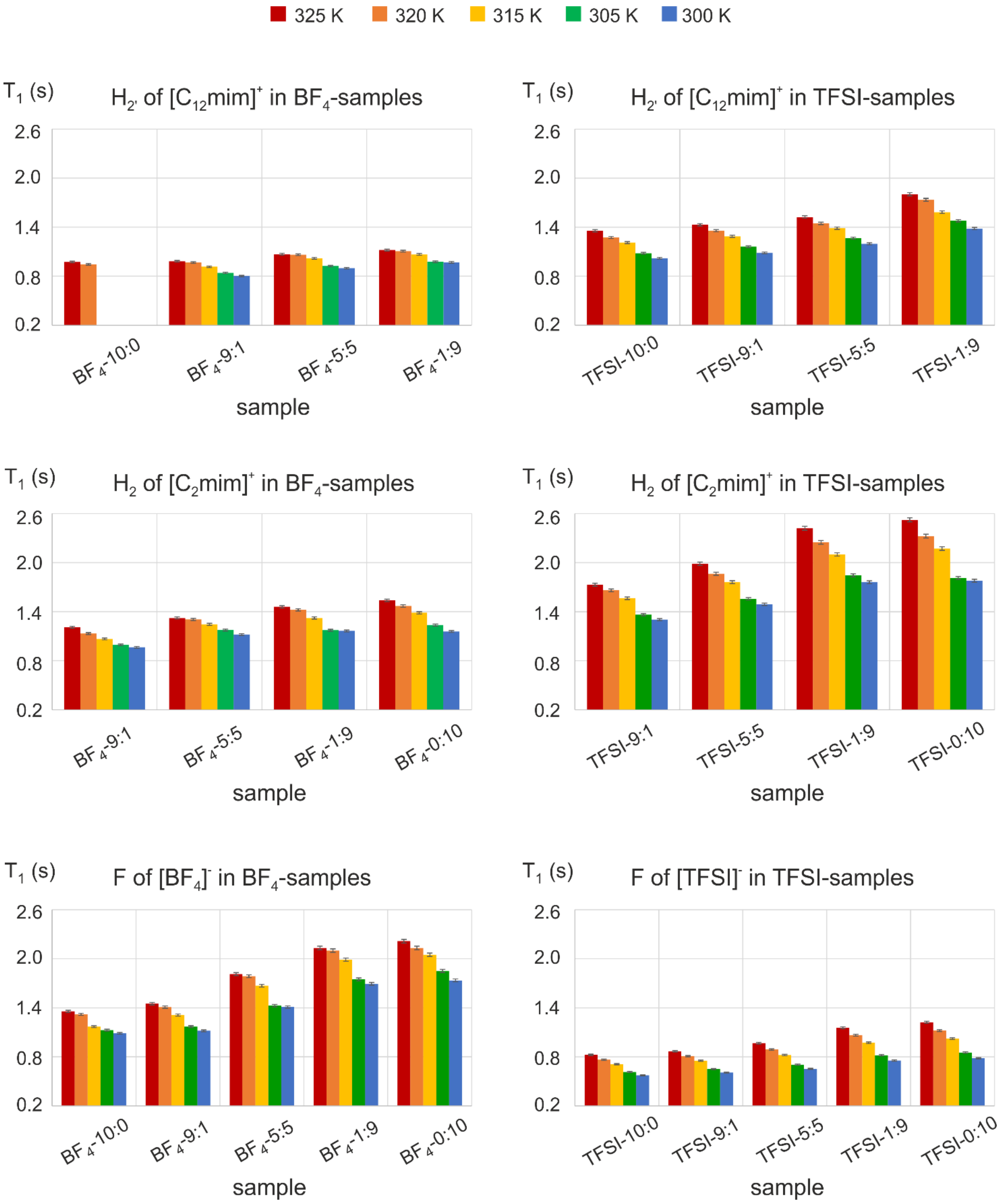

Figure S3. T_1 relaxation times measured for protons $H_{2'}$ and $H_{2'}$ of the cations $[C_{12}mim]^+$ and $[C_2mim]^+$ and for fluorine of the anion $[BF_4]^-$ or $[TFSI]^-$ in all samples. T_1 are estimated to be accurate within ±1%.

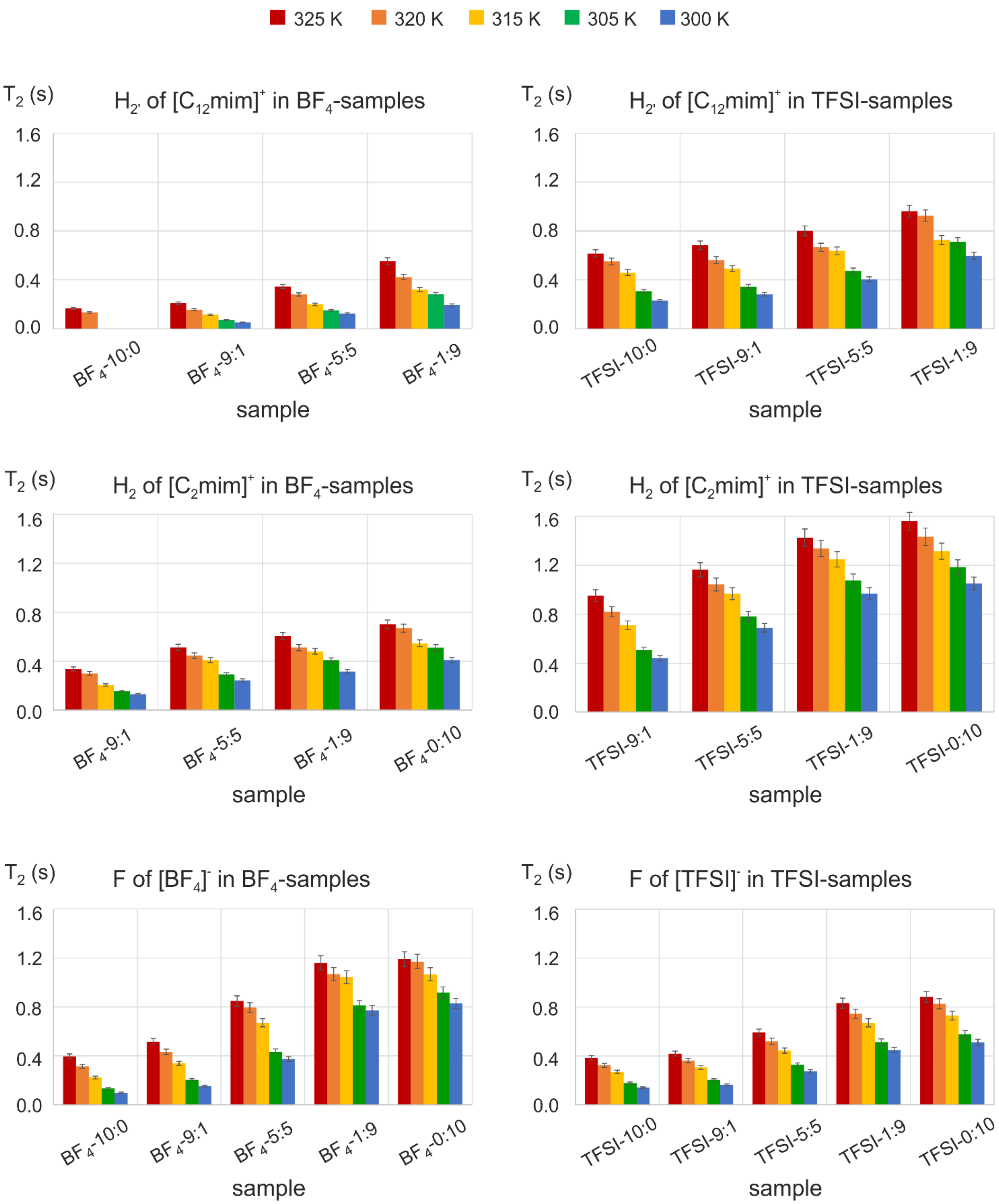

Figure S4. T_2 relaxation times measured for protons $H_{2'}$ and H_2 of the cations $[C_{12}mim]^+$ and $[C_2mim]^+$ and for fluorine of the anion $[BF_4]^-$ or $[TFSI]^-$ in all samples. T_2 are estimated to be accurate within ±5%.

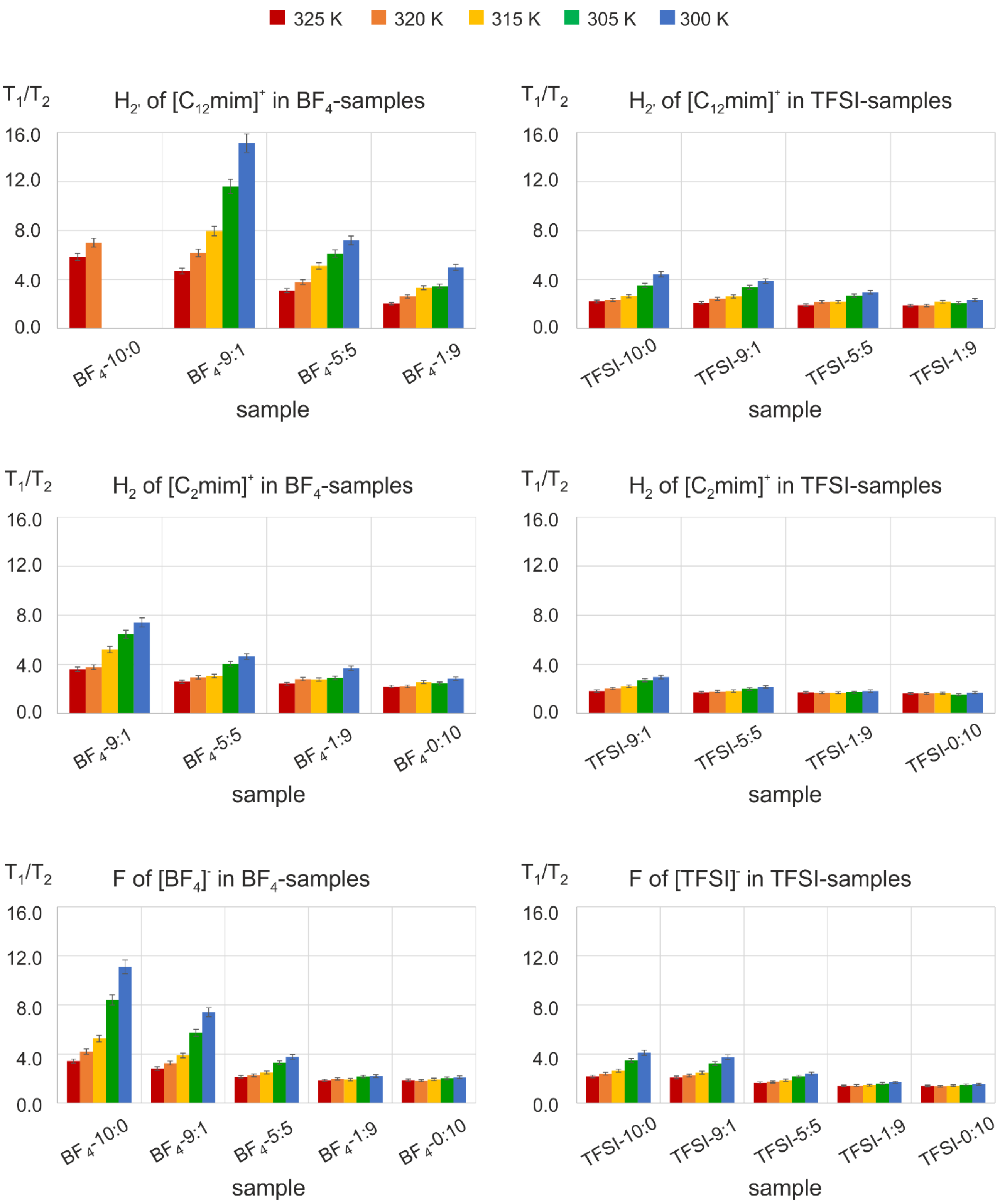

Figure S5. T_1/T_2 ratios calculated for protons $H_{2'}$ and H_2 of the cations $[C_{12}mim]^+$ and $[C_2mim]^+$ and for fluorine of the anion $[BF_4]^-$ or $[TFSI]^-$ in all samples. T_1/T_2 are estimated to be accurate within ±5%.

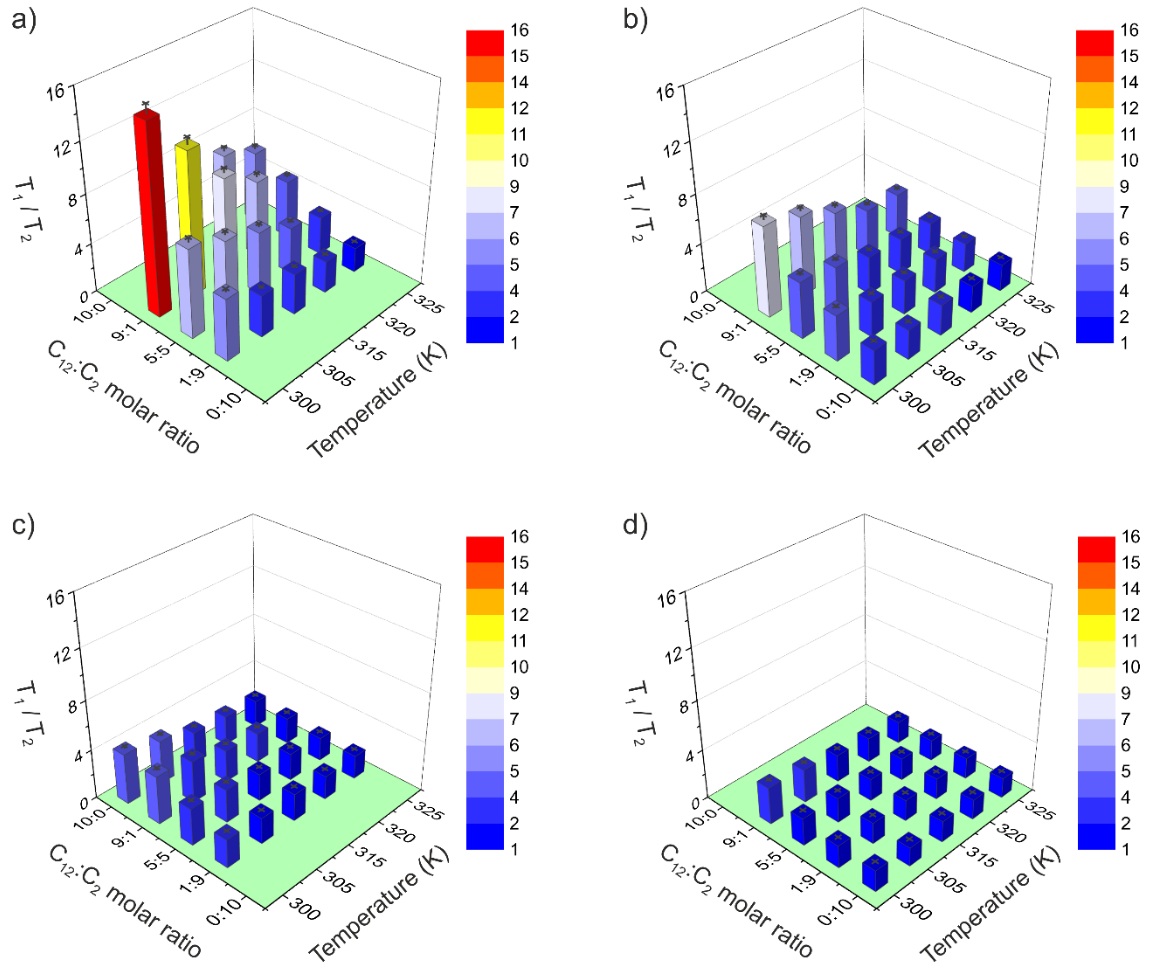

Figure S6. T_1/T_2 ratios calculated for (a) the $H_{2'}$ proton of $[C_{12}mim]^+$ in BF_4-samples, (b) the H_2 proton of $[C_2mim]^+$ in BF_4-samples, (c) the $H_{2'}$ proton of $[C_{12}mim]^+$ in TFSI-samples, and (d) the H_2 proton of $[C_2mim]^+$ in [TFSI]-samples. T_1/T_2 are estimated to be accurate within ±5%. Note that for the ionic liquid crystal $[C_{12}mim][BF_4]$ in (a) the proton relaxation times could be measured only in the isotropic range (325 K and 320 K).

Table S4. T_1 relaxation times (in s) measured for proton H_7 of $[C_2mim]^+$ and proton $H_{17'}$ of $[C_{12}mim]^+$ in all samples. T_1 are estimated to be accurate within ±1%.

	**$H_{17'}$ of $[C_{12}mim]^+$**							
**T (K)**	**BF_4-10:0**	**BF_4-9:1**	**BF_4-5:5**	**BF_4-1:9**	**TFSI-10:0**	**TFSI-9:1**	**TFSI-5:5**	**TFSI-1:9**
**325**	0.556	1.40	1.56	1.58	1.36	1.49	1.52	1.78
**320**	1.34	1.30	1.55	1.54	1.30	1.38	1.40	1.64
**315**		1.15	1.42	1.37	1.14	1.25	1.29	1.51
**305**		0.94	1.12	1.15	0.97	1.06	1.10	1.27
**300**		0.86	1.07	1.06	0.90	0.99	1.02	1.19
	**H_7 of $[C_2mim]^+$**							
**T (K)**	**BF_4-9:1**	**BF_4-5:5**	**BF_4-1:9**	**BF_4-0:10**	**TFSI-9:1**	**TFSI-5:5**	**TFSI-1:9**	**TFSI-0:10**
**325**	[a]	1.22	1.50	1.57	1.18	1.48	1.75	1.85
**320**	[a]	1.19	1.42	1.46	1.05	1.36	1.61	1.67
**315**	[a]	1.07	1.29	1.35	0.97	1.26	1.47	1.49
**305**	[a]	0.94	1.07	1.13	0.90	1.05	1.24	1.05
**300**	[a]	0.86	1.01	1.01	0.80	0.97	1.13	0.91

[a] Values not available

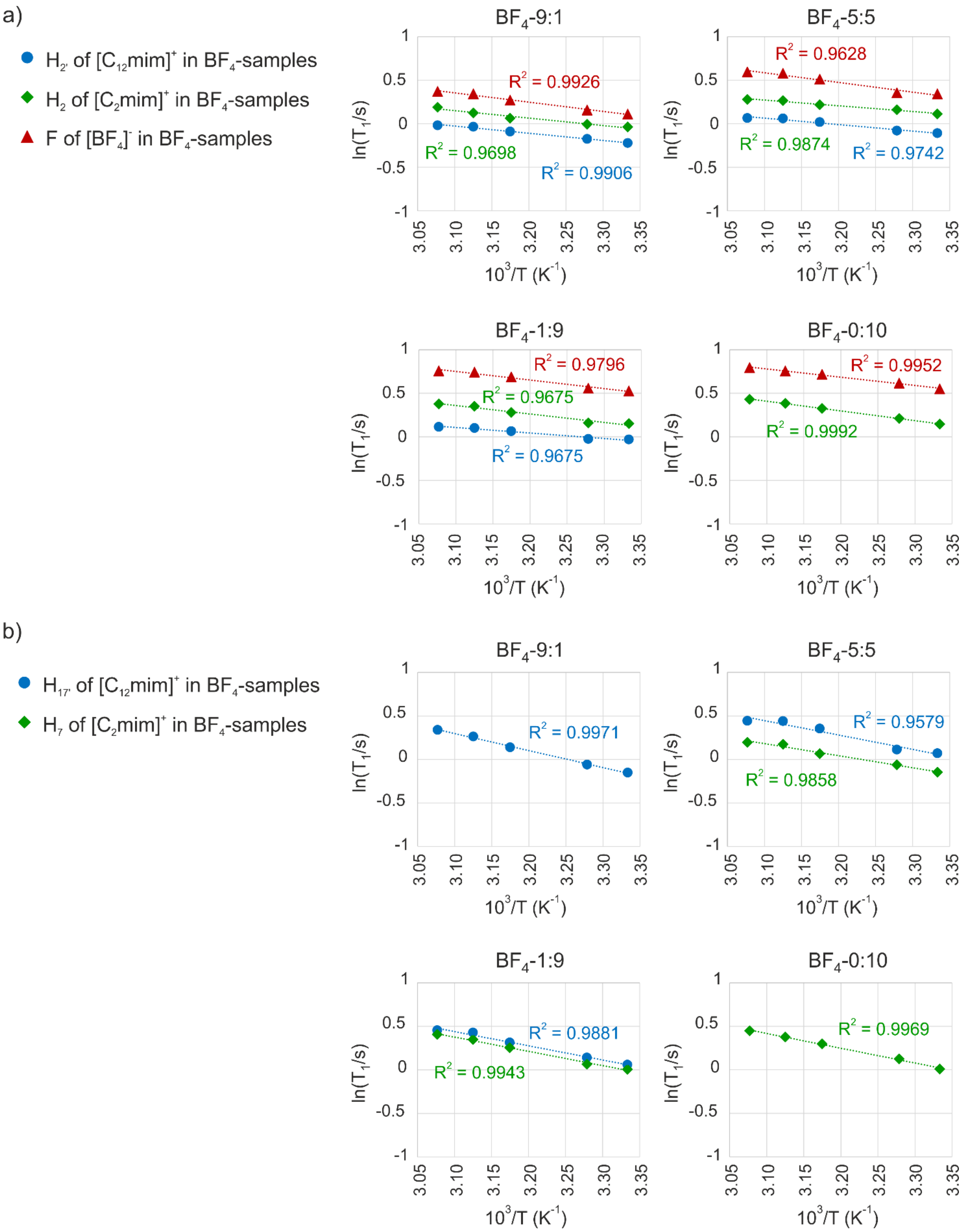

Figure S7. Arrhenius plots showing the temperature dependency of the T_1 relaxation times of (a) proton H_2 of $[C_2mim]^+$, proton $H_{2'}$ of $[C_{12}mim]^+$, and ^{19}F of $[BF_4]^-$, and (b) proton H_7 of $[C_2mim]^+$ and proton $H_{17'}$ of $[C_{12}mim]^+$ in BF_4-mixtures.

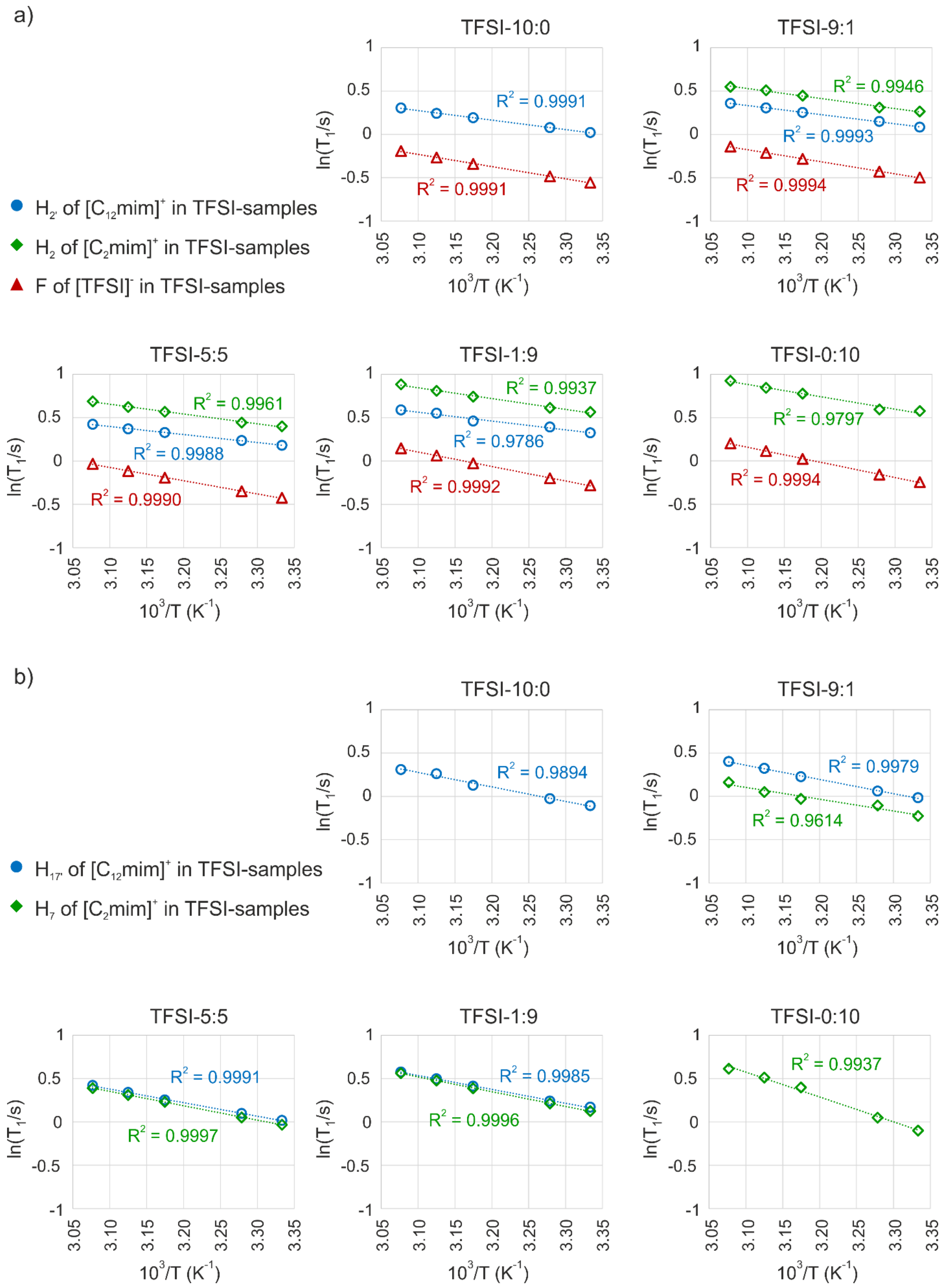

Figure S8. Arrhenius plots showing the temperature dependency of the T_1 relaxation times of (a) proton H_2 of $[C_2mim]^+$, proton $H_{2'}$ of $[C_{12}mim]^+$, and ^{19}F of $[TFSI]^-$, and (b) proton H_7 of $[C_2mim]^+$ and proton $H_{17'}$ of $[C_{12}mim]^+$ in TFSI-mixtures.

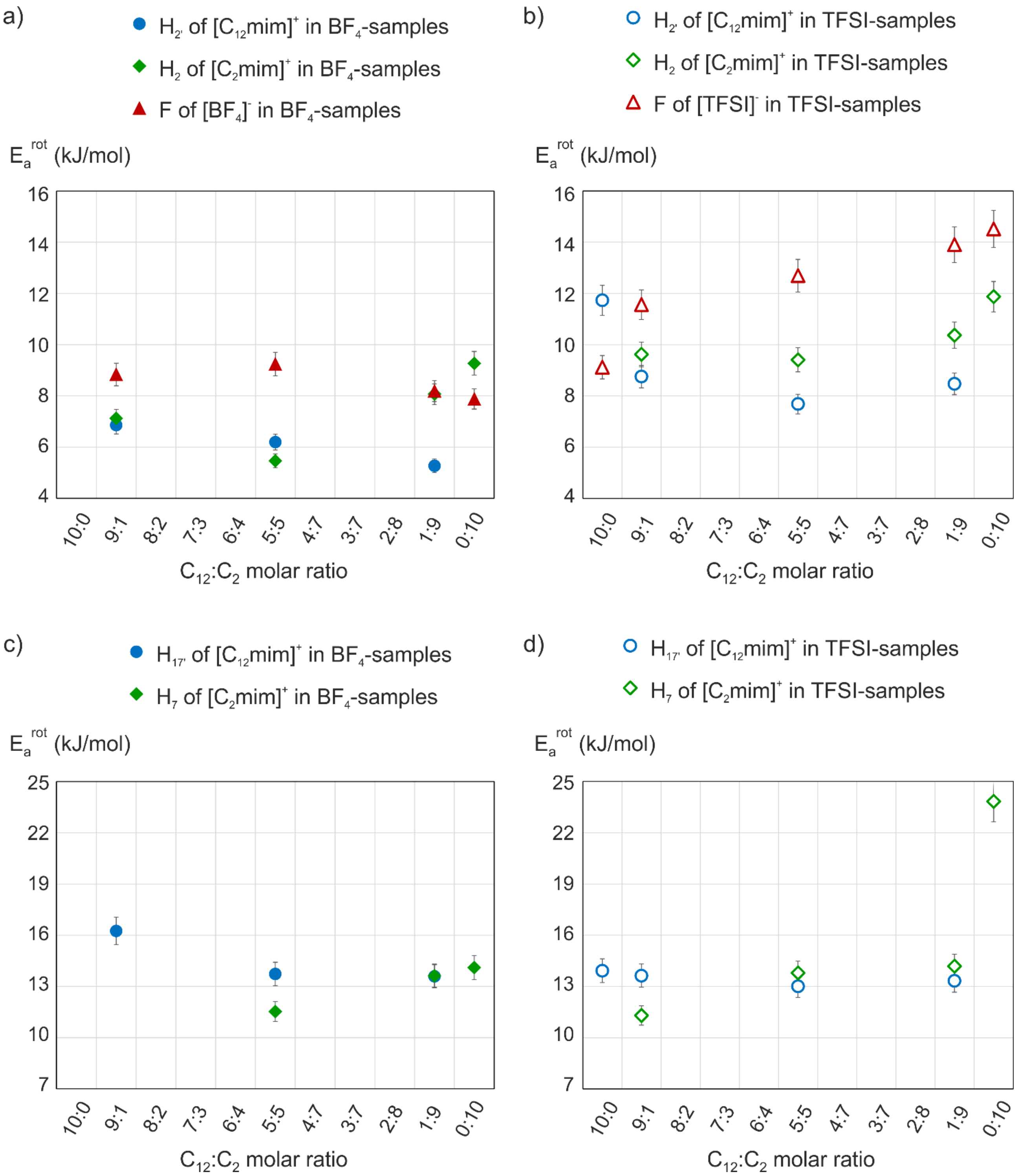

Figure S9. Apparent activation energies E_a (kJmol^{-1}) obtained from temperature-dependent T_1 data of proton H_2 of $[C_2mim]^+$, proton $H_{2'}$ of $[C_{12}mim]^+$, and ^{19}F of $[BF_4]^-$ and $[TFSI]^-$ in (a) BF_4-mixtures and (b) TFSI-mixtures. Apparent activation energies E_a (kJmol^{-1}) obtained from temperature-dependent T_1 data of proton H_7 of $[C_2mim]^+$ and proton $H_{17'}$ of $[C_{12}mim]^+$ in (c) BF_4-mixtures and (d) TFSI-mixtures. E_a are estimated to be accurate within ±5%.

Table S5. Self-diffusion coefficients measured for the cation $[C_{12}mim]^+$ in all samples. D are estimated to be accurate within ±5%.

	**D (m^2s^{-1})**							
**T (K)**	**BF_4-10:0**	**BF_4-9:1**	**BF_4-5:5**	**BF_4-1:9**	**TFSI-10:0**	**TFSI-9:1**	**TFSI-5:5**	**TFSI-1:9**
**325**	8.22E-12	1.03E-11	1.67E-11	4.91E-11	2.77E-11	2.99E-11	5.22E-11	1.09E-10
**320**	5.92E-12	8.39E-12	1.42E-11	3.63E-11	2.13E-11	2.42E-11	3.99E-11	8.58E-11
**315**		6.03E-12	1.07E-11	2.74E-11	1.64E-11	1.85E-11	2.92E-11	5.03E-11
**305**		3.37E-12	5.94E-12	1.57E-11	9.61E-12	1.10E-11	1.80E-11	3.40E-11
**300**		2.05E-12	4.82E-12	1.28E-11	7.20E-12	8.31E-12	1.46E-11	2.82E-11

Table S6. Self-diffusion coefficients measured for the cation $[C_2mim]^+$ in all samples. D are estimated to be accurate within ±5%.

	**D (m^2s^{-1})**							
**T (K)**	**BF_4-9:1**	**BF_4-5:5**	**BF_4-1:9**	**BF_4-0:10**	**TFSI-9:1**	**TFSI-5:5**	**TFSI-1:9**	**TFSI-0:10**
**325**	2.14E-11	4.62E-11	1.18E-10	1.98E-10	4.42E-11	7.90E-11	1.47E-10	1.88E-10
**320**	1.69E-11	3.98E-11	9.81E-11	1.76E-10	3.71E-11	6.37E-11	1.23E-10	1.31E-10
**315**	1.16E-11	3.17E-11	8.00E-11	1.17E-10	2.90E-11	4.92E-11	9.16E-11	1.14E-10
**305**	6.44E-12	1.86E-11	5.35E-11	7.28E-11	1.74E-11	3.18E-11	6.10E-11	7.27E-11
**300**	3.74E-12	1.48E-11	4.36E-11	6.03E-11	1.28E-11	2.52E-11	5.12E-11	5.59E-11

Table S7. Self-diffusion coefficients measured for the anions $[BF_4]^-$ or $[TFSI]^-$ in all samples. D are estimated to be accurate within ±5%.

	**D (m^2s^{-1})**				
**T (K)**	**BF_4-10:0**	**BF_4-9:1**	**BF_4-5:5**	**BF_4-1:9**	**BF_4-0:10**
**325**	9.06E-12	1.76E-11	2.66E-11	1.10E-10	1.69E-10
**320**	9.36E-12	1.43E-11	2.37E-11	8.30E-11	1.66E-10
**315**	8.58E-12	1.04E-11	1.89E-11	6.74E-11	1.02E-10
**305**	4.67E-12	5.79E-12	1.09E-11	4.39E-11	6.27E-11
**300**	3.34E-12	3.50E-12	8.39E-12	3.58E-11	5.06E-11
**T (K)**	**TFSI-10:0**	**TFSI-9:1**	**TFSI-5:5**	**TFSI-1:9**	**TFSI-0:10**
**325**	3.32E-11	3.51E-11	6.01E-11	1.20E-10	1.55E-10
**320**	2.49E-11	2.83E-11	4.69E-11	9.78E-11	9.50E-11
**315**	1.91E-11	2.17E-11	3.56E-11	5.98E-11	7.88E-11
**305**	1.13E-11	1.29E-11	2.18E-11	3.72E-11	4.47E-11
**300**	8.39E-12	9.98E-12	1.72E-11	3.19E-11	3.43E-11

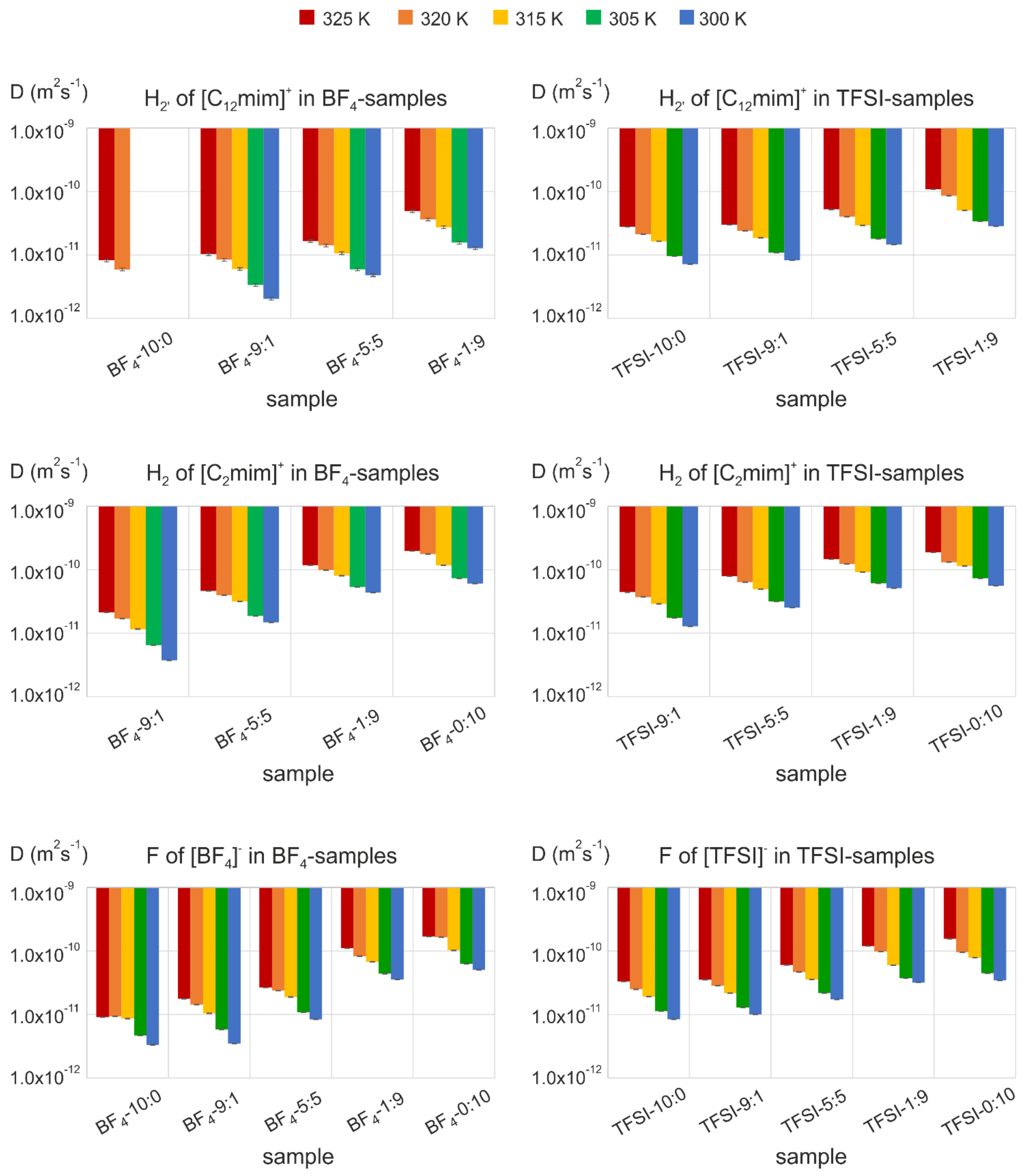

Figure S10. Diffusion coefficients measured for the cations $[C_{12}mim]^+$ and $[C_2mim]^+$ and the anion $[BF_4]^-$ or $[TFSI]^-$ in all samples. D are estimated to be accurate within ±5%.

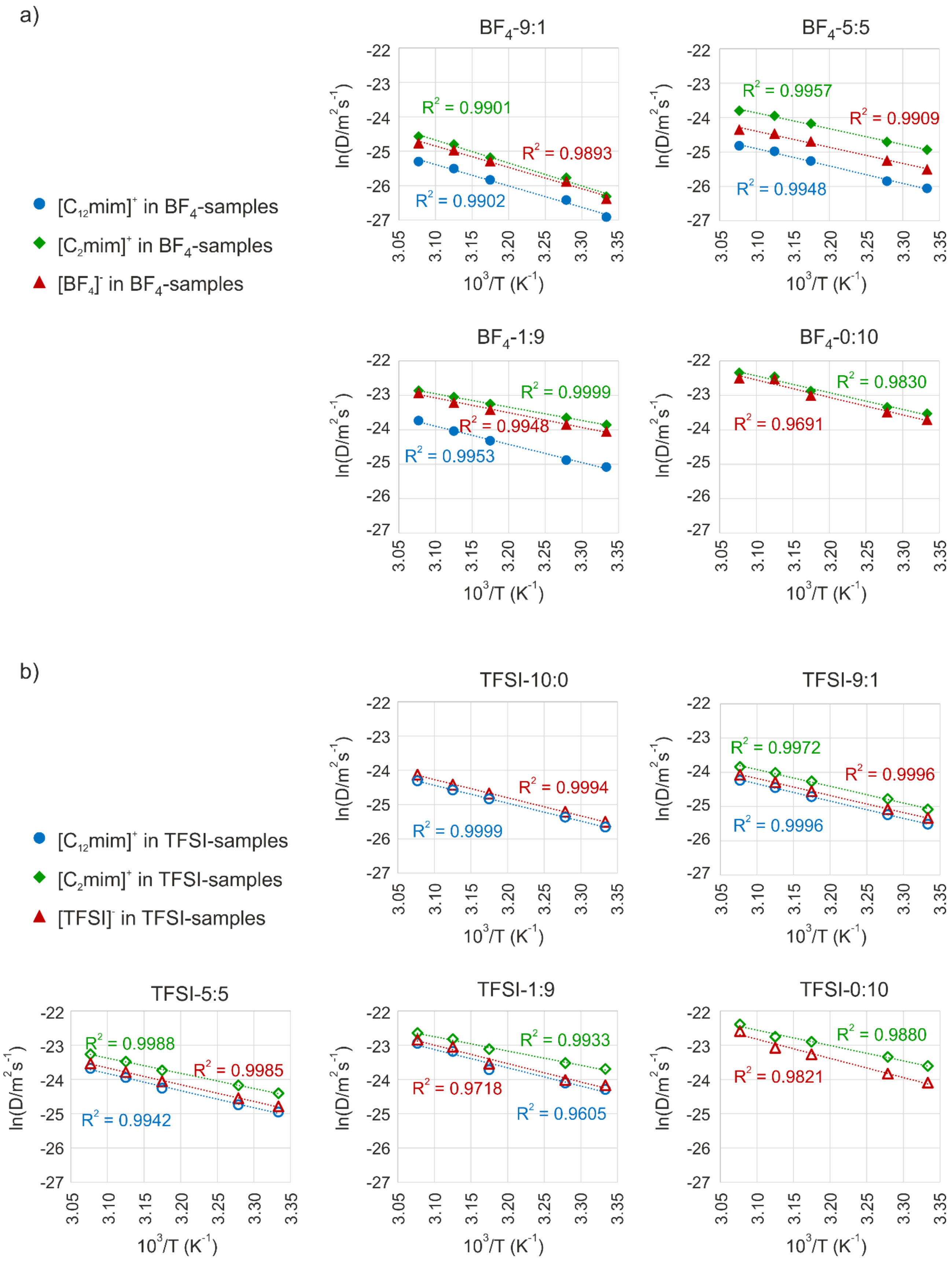

Figure S11. Arrhenius plots showing the temperature dependency of the diffusion coefficients of (a) the cations $[C_{12}mim]^+$ and $[C_2mim]^+$ and the anion $[BF_4]^-$ in BF_4-mixtures, and (b) the cations $[C_{12}mim]^+$ and $[C_2mim]^+$ and the anion $[TFSI]^-$ in TFSI-mixtures.

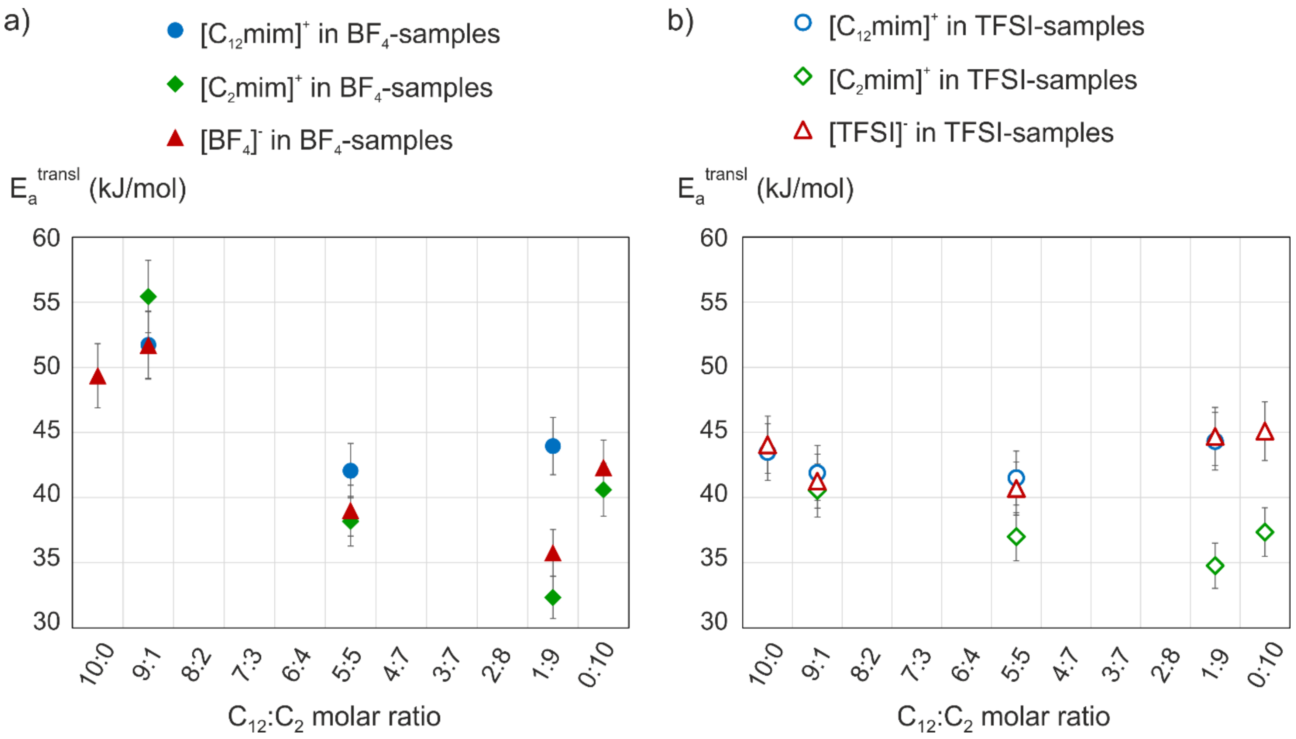

Figure S12. Apparent activation energies E_a (kJmol^{-1}) obtained from temperature-dependent diffusion data for the pure ILs and their mixtures. E_a are estimated to be accurate within ±5%.

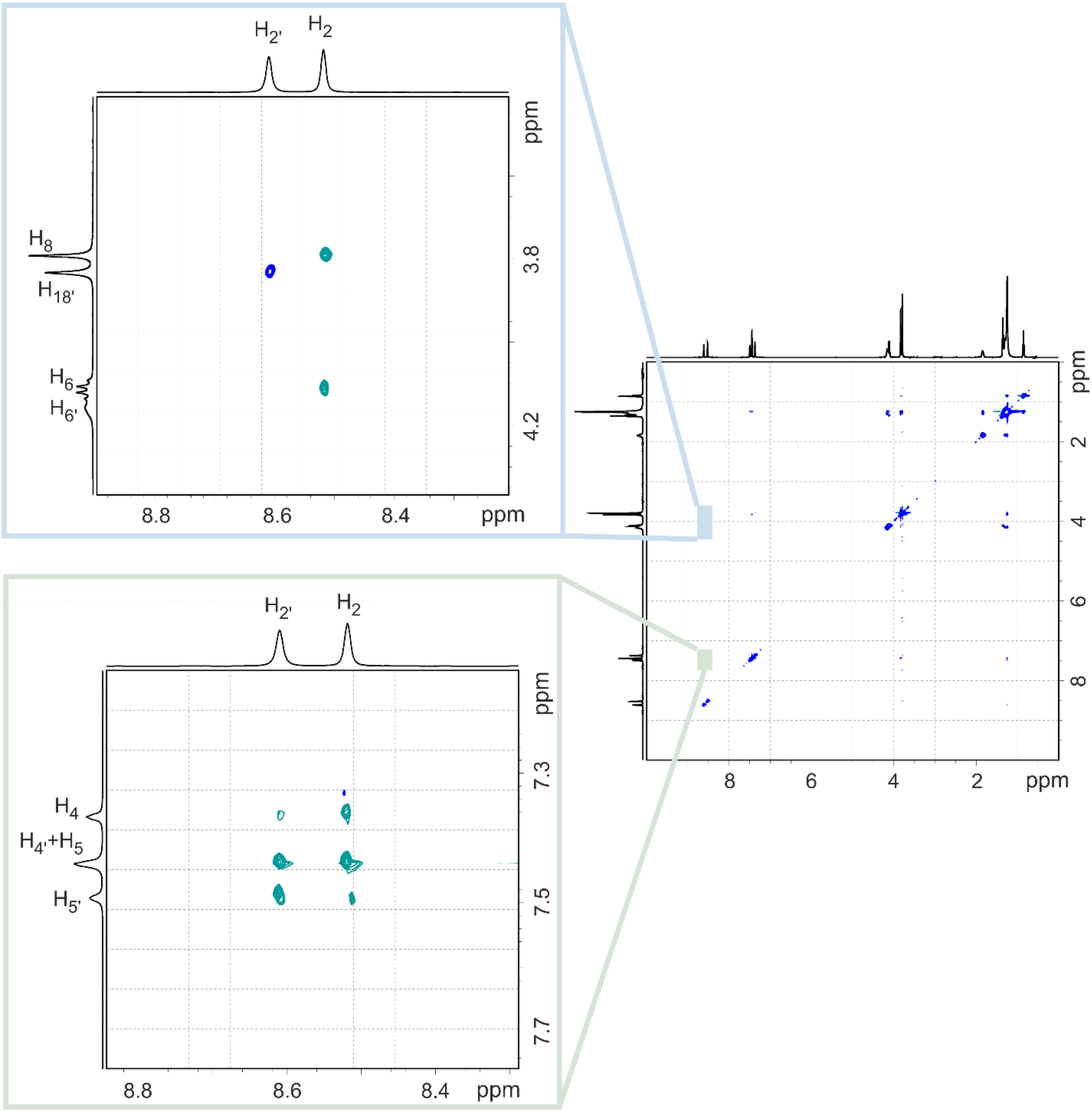

Figure S13. ROESY spectrum of BF_4-5:5 at 305 K.

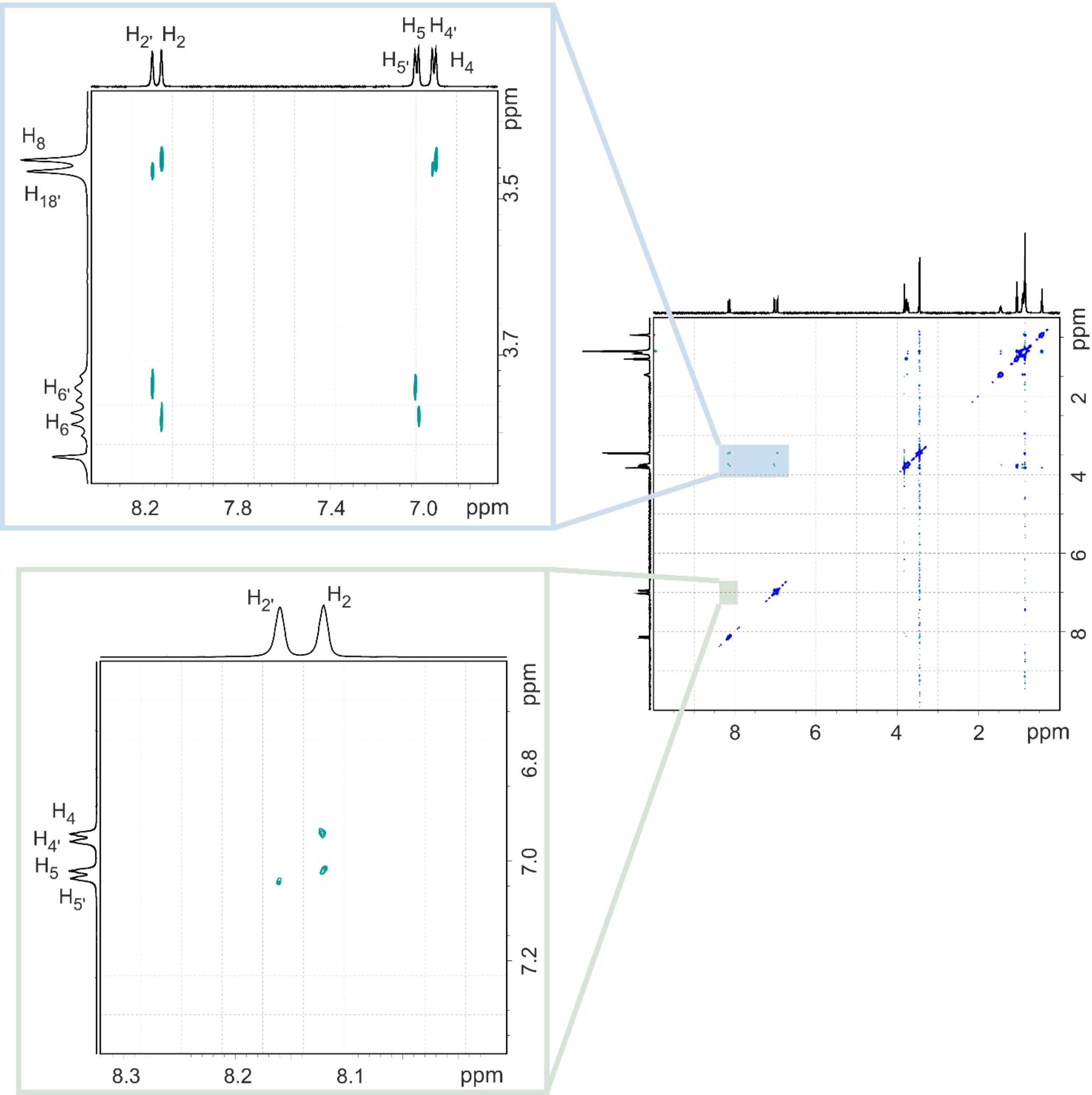

Figure S14. ROESY spectrum of TFSI-5:5 at 305 K.

Table S8. Integrated peak volume of all observed ^1H-^{19}F NOE cross peaks of sample BF_4-5:5 at 50 ms mixing time. n_H and n_F are the number of proton and fluorine nuclei contributing to the observed NOE signal. I_{raw} is the integrated volume analysed by TopSpin 4.0.3 and I_{corr} is the corrected integrals normalized by the factor $n_Hn_F/(n_H+n_F)$.

H_i	I_{raw}	n_H	n_F	$n_Hn_F/(n_H+n_F)$	I_{corr}
$H_{2'}$	1.00 [a]	1	4	0.8	1.25
H_2	0.50	1	4	0.8	0.63
$H_{5'}$	0.64	1	4	0.8	0.80
H_5 + $H_{4'}$	0.86	2	4	1.3	0.64
H_4	0.28	1	4	0.8	0.35
$H_{6'}$ + H_6	1.33	4	4	2.0	0.67
$H_{18'}$	1.27	3	4	1.7	0.74
H_8	0.57	3	4	1.7	0.33
$H_{7'}$	0.55	2	4	1.3	0.41
H_7 + $H_{8'}$-$H_{16'}$	2.16	21	4	3.4	0.64
$H_{17'}$	0.09	3	4	1.7	0.05

[a] The volume of the cross peak between $H_{2'}$ of the cation $[C_{12}mim]^+$ and the fluorines of the anion $[BF_4]^-$ was arbitrarily set to 1.

Table S9. Integrated peak volume of all observed ^1H-^{19}F NOE cross peaks of sample TFSI-5:5 at 50 ms mixing time. n_H and n_F are the number of proton and fluorine nuclei contributing to the observed NOE signal. I_{raw} is the integrated volume analysed by TopSpin 4.0.3 and I_{corr} is the corrected integrals normalized by the factor $n_Hn_F/(n_H+n_F)$.

H_i	I_{raw}	n_H	n_F	$n_Hn_F/(n_H+n_F)$	I_{corr}
$H_{2'}$	1.00 [a]	1	6	0.9	1.17
H_2	0.53	1	6	0.9	0.62
$H_{5'}$+ H_5	1.66	2	6	1.5	1.11
$H_{4'}$ + H_4	1.10	2	6	1.5	0.73
$H_{6'}$ + H_6	3.71	4	6	2.4	1.55
$H_{18'}$ + H_8	4.01	6	6	3.0	1.34
$H_{7'}$	2.01	2	6	1.5	1.34
H_7	0.65	3	6	2.0	0.33
$H_{8'}$-$H_{16'}$	21.92	18	6	4.5	4.87
$H_{17'}$	0.85	3	6	2.0	0.42

[a] The volume of the cross peak between $H_{2'}$ of the cation $[C_{12}mim]^+$ and the fluorines of the anion $[TFSI]^-$ was arbitrarily set to 1.

Theoretical background

Relaxation rates and activation energy from relaxation times

The homonuclear relaxation rates (R_1, R_2) are described using the following equations for the dipolar relaxation mechanism:[4]

$$\mathrm{R}_1 = \frac{1}{\mathrm{T}_1} = \frac{2}{5}\frac{\gamma_i^4 h^2 \mathrm{I}(\mathrm{I}+1)}{4\pi^2 r_{ij}^6}\left[\frac{\tau_C}{1+\omega_0^2\tau_C^2} + \frac{4\tau_C}{1+4\omega_0^2\tau_C^2}\right] \tag{S1}$$

$$\mathrm{R}_2 = \frac{1}{\mathrm{T}_2} = \frac{1}{5}\frac{\gamma_i^2 h^2 \mathrm{I}(\mathrm{I}+1)}{4\pi^2 r_{ij}^6}\left[\frac{5\tau_C}{1+\omega_0^2\tau_C^2} + \frac{2\tau_C}{1+4\omega_0^2\tau_C^2} + 3\tau_C\right] \tag{S2}$$

where γ is the ^{1}H or ^{19}F gyromagnetic ratio, h is Planck's constant, I is the spin quantum number, r_{ij} is the inter-proton (-fluorine) distance, τ_C is the correlation time for molecular reorientation and ω_0 is the ^{1}H or ^{19}F Larmor frequency.

The relaxation rate $1/\mathrm{T}_1$ is proportional to τ_C in the region where $\omega_0\tau_C \ll 1$ (extreme narrowing condition) and is proportional to $1/\tau_C$ in the region where $\omega_0\tau_C \gg 1$. This corresponds to curves for T_1 vs temperature with a positive slope in the higher temperature region / lower viscosity and a negative slope in the lower temperature region / higher viscosity, with the minimum value at $\omega_0\tau_C = 0.62$.

The relaxation rate $1/\mathrm{T}_2$ is mainly proportional to τ_C in Eq (S2), then T_2 values simply decrease with decreasing temperature.

The temperature-dependent correlation time τ_C obeys the Arrhenius equation:[5]

$$\tau_C = \tau_A\, exp\left(-\frac{E_a}{RT}\right) \tag{S3}$$

where E_a is the activation energy, R is the gas constant, and T is the absolute temperature.

For $\omega_0\tau_C \ll 1$, Eq (S3) can be rewritten as:[5]

$$\mathrm{T}_1 = \mathrm{T}_{1,\mathrm{A}}\, exp\left(-\frac{E_a}{RT}\right) \tag{S4}$$

Therefore, in the extreme narrowing limit the activation energy of rotational motion can be estimated from the temperature dependence of the T_1 values.

Self-diffusion and activation energy from diffusion coefficients

In a BPP-LED experiment, the measurement of the diffusion coefficients D is achieved by fitting the observed echo intensity I by the Stejskal-Tanner equation:[6]

$$I = I_0\, exp\left[-(\gamma g \delta)^2 D\left(\Delta - \frac{\delta}{3}\right)\right] \tag{S5}$$

where I_0 is the echo intensity without field gradient, γ is the gyromagnetic ratio of the observed nucleus, and g is the maximum magnetic field gradient strength.

The temperature dependence of the self-diffusion can be fitted with the exponential Arrhenius equation to obtain the activation energy for diffusive flow, that is the amount of energy required for the molecules in the fluid mixture to diffuse:[7]

$$D(T) = D_0\, exp\left(-\frac{E_a}{RT}\right) \qquad \text{(S6)}$$

where D_0 is a preexponential factor for the diffusive flow, E_a is the activation energy, R is the gas constant, and T is the absolute temperature.

References

1. Bruce DW, Cabry CP, Lopes JNC, et al. Nanosegregation and Structuring in the Bulk and at the Surface of Ionic-Liquid Mixtures. *J Phys Chem B*. 2017;121(24):6002-6020. doi:10.1021/acs.jpcb.7b01654
2. Navia P, Troncoso J, Romaní L. Viscosities for ionic liquid binary mixtures with a common ion. *J Solution Chem*. 2008;37(5):677-688. doi:10.1007/s10953-008-9260-8
3. Song D, Chen J. Density and viscosity data for mixtures of ionic liquids with a common anion. *J Chem Eng Data*. 2014;59(2):257-262. doi:10.1021/je400332j
4. Solomon I. Relaxation processes in a system of two spins. *Phys Rev*. 1955;99(2):559-565. doi:10.1103/PhysRev.99.559
5. Shimizu Y, Wachi Y, Fujii K, Imanari M, Nishikawa K. NMR Study on Ion Dynamics and Phase Behavior of a Piperidinium-Based Room-Temperature Ionic Liquid: 1-Butyl-1-methylpiperidinium Bis(fluorosulfonyl)amide. *J Phys Chem B*. 2016;120(25):5710-5719. doi:10.1021/acs.jpcb.6b04095
6. Castiglione F, Moreno M, Raos G, et al. Structural organization and transport properties of novel pyrrolidinium-based ionic liquids with perfluoroalkyl sulfonylimide anions. *J Phys Chem B*. 2009;113(31):10750-10759. doi:10.1021/jp811434e
7. Castiglione F, Raos G, Appetecchi GB, et al. Blending ionic liquids: how physico-chemical properties change. *Phys Chem Chem Phys*. 2010;12(8):1784-1792. doi:10.1039/c001176m
